# Metal Additive Manufacturing for Automotive Applications: Technologies, Materials, Industrial Uses, and Future Directions

**DOI:** 10.3390/ma19214635

**Published:** 2026-10-31

**Authors:** Álvaro Rodríguez-Díaz, Eneko Villabona, Juan Sustacha, Tomas Ballesteros, Virginia Uralde, Fernando Veiga

**Affiliations:** 1 Department of Engineering, Campus of Arrosadía, Public University of Navarre, 31006 Pamplona, Spain; ard@edu.xunta.gal (Á.R.-D.); sustacha.174559@e.unavarra.es (J.S.); tomas.ballesteros@unavarra.es (T.B.); 2 Department of Transportation and Maintenance of Vehicles, CIFP Fontecarmoa, 36600 Vilagarcía de Arousa, Spain; 3 Department of Engineering, Campus of Tudela, Public University of Navarre, 31500 Tudela, Spain; virginia.uralde@unavarra.es

**Keywords:** metal additive manufacturing, automotive industry, LPBF, binder jetting, directed energy deposition, WAAM, metal material extrusion, tooling, spare parts, lightweight design

## Abstract

Metal additive manufacturing (MAM) offers automotive manufacturers design and supply-chain options, but published demonstrations do not by themselves establish production readiness or economic advantage. This critical narrative review connects process selection, alloy behaviour, component requirements and the complete manufacturing chain. It covers laser powder bed fusion, directed energy deposition, binder jetting and metal material extrusion, distinguishing direct vehicle parts from tooling, repair and spare parts. The synthesis identifies the following three recurring constraints: fatigue performance cannot be inferred from tensile strength; sintering-based routes require control of shrinkage and local defects; and printing productivity must be separated from output of accepted finished parts. Published examples include a 10% piston mass reduction and a 17% moulding-cycle reduction, both reported by industrial sources rather than established as sector-wide effects. A requirement-led selection framework, condition-specific material benchmarks and a qualitative economic assessment connect these findings to engineering decisions. The strongest opportunities occur where functional integration, avoided tooling or asset-life extension offset qualification and post-processing costs. Evidence remains heterogeneous and the initial single-database search limits coverage; the review therefore does not claim a systematic census or a universal ranking of technologies. Adoption should be judged at the level of a qualified alloy–process–part combination against a credible conventional alternative.

## 1. Introduction

The automotive industry is under simultaneous pressure to shorten development cycles, diversify vehicle architectures, reduce mass, improve supply-chain resilience and accelerate the transition toward electrified and more sustainable products. In this context, additive manufacturing (AM) has evolved from a rapid prototyping tool into a broader family of manufacturing routes that can support design validation, production tooling, spare-part supply, functional testing and, in selected cases, end-use metallic components [1,2,3,4]. Within AM, metallic processes are especially relevant when the target application requires structural integrity, thermal resistance, wear performance or compatibility with established engineering alloys.

The main industrial attraction of metal additive manufacturing (MAM) is not only geometric freedom. Its real value lies in the combination of freedom of design, part consolidation, local customisation, shorter lead times, distributed production and the possibility of integrating digital engineering directly with manufacturing [1,4]. For automotive companies, this combination is meaningful in at least four recurring scenarios as follows: (i) prototypes and pre-series components with short lead-time constraints; (ii) high-value or low-volume vehicle parts where tooling amortisation is difficult; (iii) production aids such as jigs, fixtures and inserts that benefit from fast iteration; and (iv) aftersales, legacy and repair applications where conventional supply is inefficient [1,3,4].

Industrial reports document both deployed applications and development programmes. BMW reports more than 1.6 million components produced at its Additive Manufacturing Campus since operations began and more than 100,000 produced annually at decentralised plants [5]. These totals encompass AM activities and must not be interpreted as counts of qualified metal end-use parts. Porsche reports a development project for printed aluminium pistons [6], while Volkswagen describes a binder-jetting industrialisation programme [7]. Each case is classified by the evidence actually reported in Section 6.

The engineering gap addressed here is how to connect a proposed automotive function with an alloy, an AM process chain, a qualification plan and a conventional cost baseline. The objectives are to distinguish demonstrated use from prospective use, identify property and manufacturing constraints, and provide decision criteria for direct components, factory tooling and lifecycle support. This is a critical review of published evidence; it does not present new manufacturing experiments or a meta-analysis.

### 1.1. Existing Reviews and Contribution of This Work

The metal automotive review [1] already covers a closely related topic, while the broader mini review [2] includes nonmetallic AM and sectoral applications. The conference review [3] also addresses applications, advantages and limitations. These studies show that neither automotive AM nor the combination of processes and applications is a new review topic.

The automotive status-and-challenges contribution [8] and the hybrid-manufacturing analysis [9] provide additional context. Process-specific reviews, such as the wire-arc synthesis [10], and material-specific reviews, such as the corrosion analysis [11], supply depth that a sector-wide review cannot reproduce for every alloy.

Table 1 compares the principal scope of these contributions with the present review. The distinction is the explicit connection between requirements, evidence quality, processing constraints, conventional alternatives and accepted-part economics. This is a difference in synthesis emphasis, not a claim that earlier papers entirely omitted these subjects.

The proposed selection framework is an engineering synthesis of the literature, not a validated predictive algorithm. Its usefulness depends on application-specific loading, quality and cost data. Purely polymeric production aids are outside the material scope even when included in a manufacturer’s overall AM statistics.

### 1.2. Process Terminology and Scope

**Figure 1:**
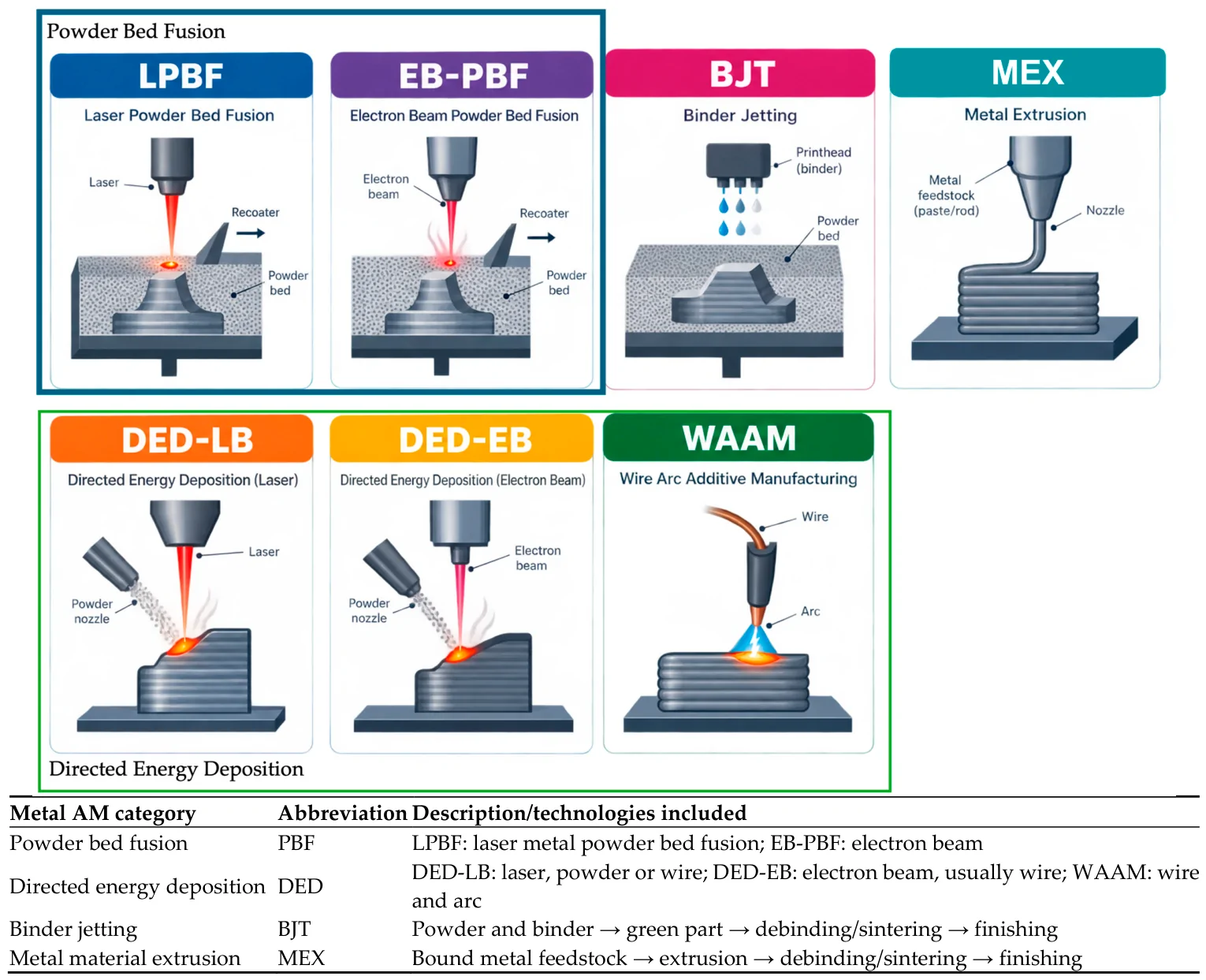
Metal AM taxonomy used in this review. WAAM is an arc-based DED route. Diagram prepared for this review from the terminology in [12].

ISO/ASTM 52900 groups additive manufacturing into seven process categories [12]. The present review focuses on powder bed fusion (PBF), directed energy deposition (DED), binder jetting (BJT) and metal material extrusion (MEX). Laser powder bed fusion is abbreviated LPBF, and electron-beam powder bed fusion is denoted EB-PBF. Laser-beam and electron-beam DED are denoted DED-LB and DED-EB, respectively. Wire-arc additive manufacturing (WAAM) is an arc-based DED route, not a separate category alongside DED. Figure 1 summarises the taxonomy; Section 3 explains the processes.

The four selected categories are not equally mature, and a category cannot be qualified independently of its alloy, machine, parameters and component requirements. Laboratory results, industrial demonstrations and reported production applications are therefore distinguished explicitly.

From an automotive engineering perspective, these technologies should not be compared only in terms of dimensional accuracy. Their industrial suitability depends on build rate, powder or wire cost, post-processing burden, design freedom, attainable part size, surface finish, reproducibility, qualification effort and total cost per accepted part. The trade-off is therefore process-chain specific: the best technology for a lightweight high-performance bracket is not necessarily the best one for a mould insert, a repaired die, or a low-cost spare part.

A second useful distinction is between single-step and multi-step AM routes [12]. LPBF and many DED routes generally produce a consolidated metallic part during the build itself, whereas binder jetting and metal material extrusion are multi-step routes that rely on downstream debinding and/or sintering to obtain final metallic properties. This distinction is highly relevant in automotive decision-making because it affects dimensional control, energy use, batch logistics, part density and scalability.

## 2. Methodology

**Figure 2:**
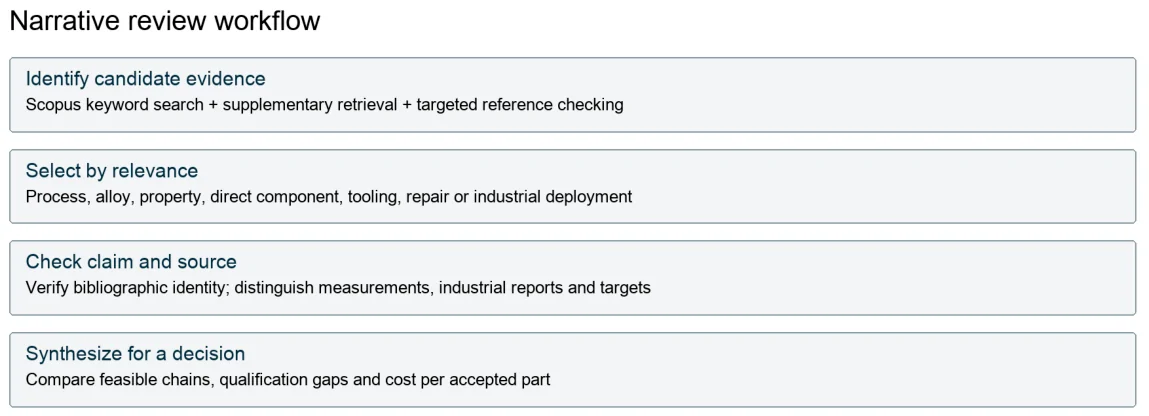
Workflow of the structured literature search, thematic screening and critical narrative synthesis.

This work follows a structured literature-search and thematic-screening approach to critically review MAM for automotive applications. The following two complementary retrieval routes were used: a conventional Scopus search on 21 May 2026 and a search using Scopus LeapSpace on 25 July 2026. Subsequent monitoring of company websites and specialist magazines supplied practical industrial cases. The selected evidence was organised by process, material, application and industrial implementation. Figure 2 summarises the synthesis workflow. The study is a critical narrative review rather than a systematic review or meta-analysis.

### 2.1. Search Strategy

**Figure 3:**
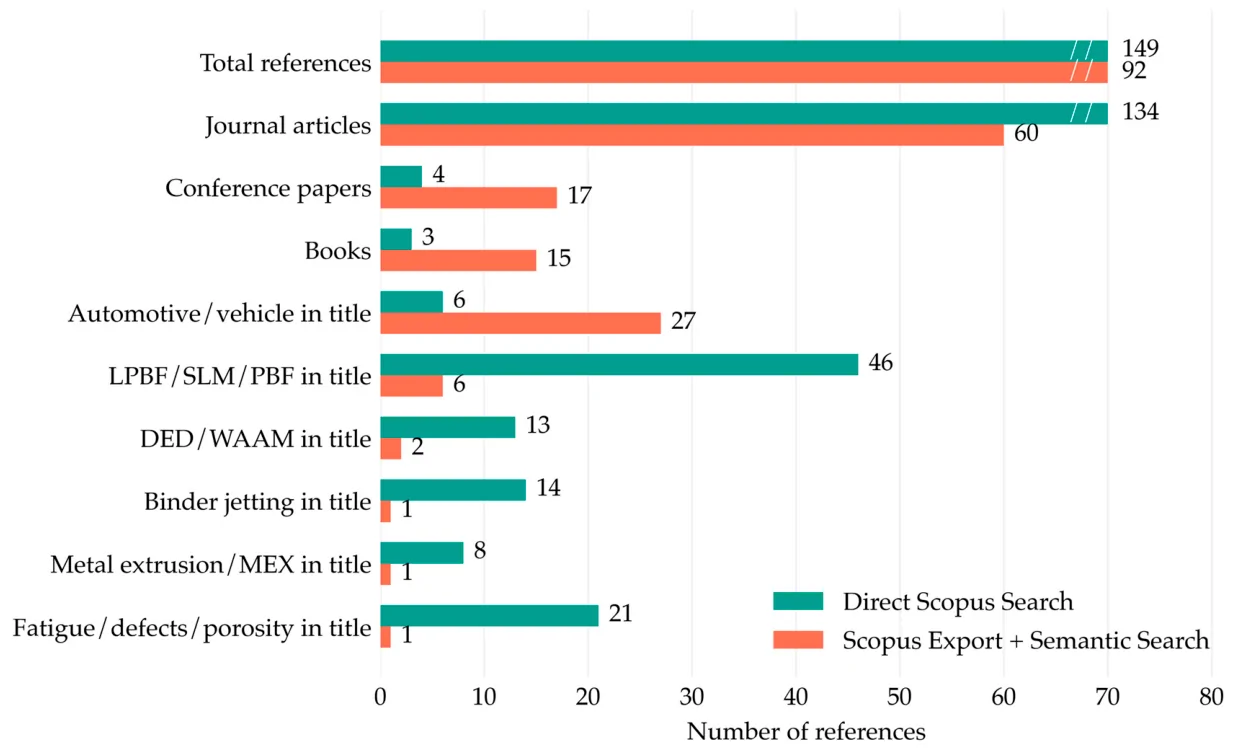
Comparison of the thematic coverage obtained using the conventional Scopus-based literature search and the Scopus export combined with Semantic Search.

The results of a conventional Scopus search combining “metal additive manufacturing” AND “automotive industry” are shown in Figure 3. Review-related records were examined first to establish the scope, including the metal automotive review [1], the broader automotive mini review [2] and the conference contribution [3]. The same terms were then used for wider scientific retrieval. The same search terms and thematic scope were used for the complementary Semantic Search. The conventional strategy provided broader process- and materials-oriented coverage, whereas Semantic Search showed a stronger concentration of publications explicitly related to automotive and vehicle applications. The thematic categories are not mutually exclusive.

The initial search favoured publications explicitly linking metal AM with automotive terminology. Such a strategy may omit process studies indexed under alloy names, laser powder bed fusion, binder jetting, wire arc deposition, tooling or repair. It may also miss SAE and FISITA proceedings, Web of Science-only records and industrial reports. Consequently, the synthesis should be interpreted as a selected evidence base rather than a representative census of all industrial activity.

Targeted supplementary searches and bibliographic checks were completed on 13 September 2026 using article titles, DOI records and publisher or manufacturer pages. These checks covered the automotive conference contribution [8], the hybrid-manufacturing study [9], recent H13 and visible-wavelength copper studies [13,14,15], and an experimental casting–LPBF comparison [16]. This was a targeted update and verification exercise, not a new systematic search in Scopus or Web of Science.

The complementary search was conducted using Scopus LeapSpace on 25 July 2026, with the same search terms and thematic scope as the conventional Scopus query. Its purpose was to identify the additional literature of conceptual relevance to automotive applications. The retrieved records were assessed using the same eligibility criteria as the conventional-search results. The two routes were used to broaden the candidate evidence base; no quantitative improvement in retrieval performance is inferred.

**Figure 4:**
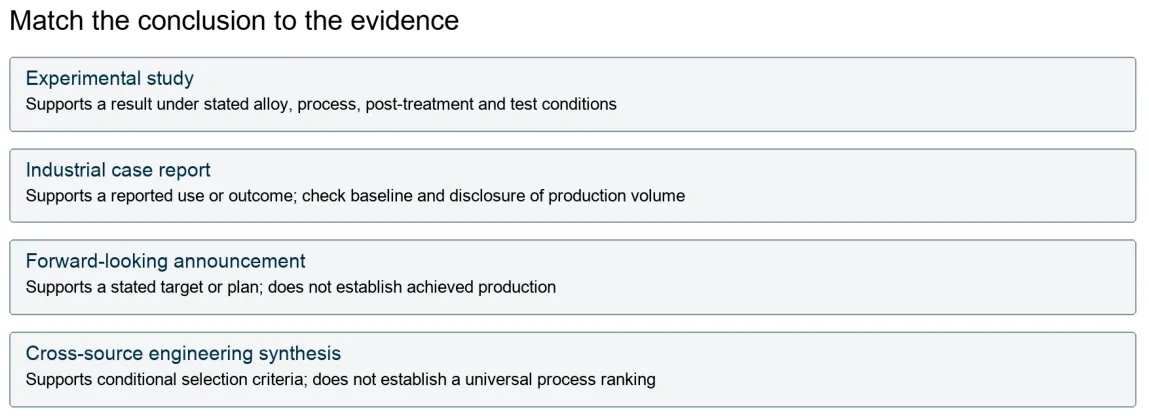
Evidence distinctions used in the synthesis. Experimental measurements, company-reported outcomes and forward-looking announcements answer different questions and are not interchangeable measures of readiness.

Practical industrial cases were subsequently identified through monitoring of company websites and specialist magazines, using company names, component terms and manufacturing technologies. Where available, reports were checked against the original manufacturer or technology-provider release. The source date, reported alloy and process, stated deployment status and available quantitative outcomes were recorded. Industrial reports were used as evidence of reported applications, distinct from peer-reviewed experimental results; they do not independently establish sector-wide performance or profitability. Figure 4 summarises these evidence distinctions.

Evidence categories are not mutually exclusive at the publication level; the category applies to the specific claim being assessed.

### 2.2. Screening and Eligibility Criteria

The conventional Scopus results were screened sequentially by removing duplicates and irrelevant document types, examining titles and abstracts, and assessing relevant full-text content against the scope of the review. Records retrieved through LeapSpace were examined using the same thematic eligibility criteria. Industrial cases were assessed for their relevance to the process, material, component and implementation questions addressed by the review.

The conventional and LeapSpace searches served complementary retrieval purposes, but their relative coverage and percentage overlap were not quantified using reconciled, deduplicated record lists. Consequently, no claim of superior retrieval performance or a measured degree of complementarity is made.

The following topics were eligible when their findings could inform an automotive manufacturing or qualification decision:

- analysis of MAM technologies with clear relevance to automotive engineering or manufacturing;
- discussion of metallic materials, microstructures, defects, or mechanical/functional properties relevant to automotive requirements;
- applications in automotive parts, components, or subsystems;
- auxiliary applications such as tooling, jigs, fixtures, moulds, inserts, spare parts, remanufacturing, or repair; and
- industrial implementation aspects such as qualification, cost, productivity, scalability, sustainability, or future deployment.

Publications were excluded when their main focus was unrelated to metallic feedstocks, when the automotive context was only incidental, when they dealt exclusively with other sectors without transferable implications, or when they did not provide useful information for the thematic objectives of this review. Studies centred only on general AM without sufficient relevance to metal processes or automotive use were also excluded.

### 2.3. Review Scope and Data Organisation

After screening, the selected publications were organised into thematic groups consistent with the structure of the review:

- definition and classification of MAM technologies;
- process families with the highest relevance for automotive applications;
- metallic materials and associated property-related considerations;
- applications in direct and auxiliary automotive components; and
- future research and industrial trends.

For each retained quantitative comparison, the relevant process, alloy, post-processing and test conditions are stated when reported. Values with incompatible stress ratios, surface conditions, temperatures or definitions of throughput are not pooled. Missing information is identified as a limitation of the comparison.

Selection bias, publication bias toward successful demonstrations and incomplete reporting of rejected parts or costs limit the synthesis. In particular, a successful coupon result does not establish component qualification, and a company target does not establish achieved serial output. These limitations constrain the maturity and economic conclusions throughout the review.

## 3. Process Principles and Capability Limits

### 3.1. Powder Bed Fusion

LPBF selectively melts successive powder layers. Its geometric capability supports internal channels, lightweight brackets and functional integration, provided that powder can be removed and critical surfaces can be inspected and finished [17,18,19]. Automotive examples include development pistons with internal cooling passages and a demonstrator titanium brake calliper [6,20]. Neither example establishes a generic process tolerance or unrestricted series-production readiness.

Numerical capability should be tied to a machine and material. A published TruPrint 5000 Green Edition specification gives a cylindrical build envelope of 300 mm diameter × 400 mm height, layer thicknesses of 30–150 μm and a build rate of 8–100 cm^3^/h including exposure and recoating; actual values depend on material, filling and configuration [21]. Layer thickness and laser spot diameter are not minimum feature size or dimensional accuracy. The Bugatti calliper demonstrates 1–4 mm walls in one particular design [20]. Supports, distortion control and machining allowances still constrain usable geometry.

LPBF consolidation depends on energy input, powder condition, scan strategy and melt-pool stability. Unstable processing can cause lack of fusion or keyhole porosity [22,23,24,25,26]. EB-PBF uses a vacuum environment and elevated powder-bed temperature; the latter can reduce thermal gradients and residual stresses and is useful for selected titanium alloys and intermetallic systems. Coarser surface texture, powder-cake removal and machine-specific alloy availability can restrict automotive deployment [27]. EB-PBF is distinct from DED-EB, in which feedstock is delivered during deposition.

### 3.2. Directed Energy Deposition

DED melts feedstock as it is supplied to a substrate. DED-LB can use powder or wire, DED-EB commonly uses wire, and WAAM uses wire and an arc [12,28,29]. Powder-fed laser deposition offers local material placement, whereas wire routes favour material delivery efficiency and larger near-net-shape structures. WAAM commonly operates on a kilogram-per-hour scale; the reviewed wire-arc literature reports approximately 1–10 kg/h, with geometry, alloy and thermal control determining the usable rate [28]. These are deposition rates, not rates of inspected finished automotive parts.

The build envelope of robotic DED is governed by the qualified cell workspace, access, shielding and thermal management rather than by a powder-bed box. Bead width and layer height set a coarser geometric scale than fine LPBF features; they should be measured for the proposed wire or powder process rather than replaced by an assumed universal resolution. Laser deposition has been investigated for restoration of stainless-steel and titanium surfaces [30]. Application to an automotive die also requires substrate-specific validation of dilution, heat-affected-zone properties and thermal fatigue. Hybrid deposition and machining can restore local features while retaining the conventionally manufactured bulk [9].

### 3.3. Binder Jetting

BJT deposits binder selectively into a powder bed and obtains final metallic consolidation through subsequent thermal processing. Its principal advantage is the separation of printing from furnace densification, which permits batch processing without melt-induced distortion during printing [31,32]. It does not eliminate thermal distortion: shrinkage and gravitational deformation occur during sintering. HP lists 316 L and 17-4PH stainless steels for its Metal Jet platform and an effective S100 print volume of 430 × 309 × 170 mm [33]. This is a green-part envelope, not the maximum final sintered part size.

No single maximum density or strength describes BJT. Powder packing, binder distribution, chemistry, furnace atmosphere, sintering temperature and local section thickness determine residual porosity and microstructural uniformity [31,34]. A comparative H13 study reported 99.3% relative density for BJT, compared with 99.9% for LPBF and greater than 99.9% for wire-laser DED under its own processing conditions [13]. These observations are not guaranteed minima. Residual pores, segregation and local gradients can remain important for fatigue even at high average density. Load-bearing use therefore requires component-representative cyclic and dimensional qualification.

Stainless steels provide commercial reference cases, while copper and other material systems require separate validation of spreading, binding and densification [33,35]. A recent BJT modelling review concentrates on green-part formation and binder–powder interactions; it should not be treated as proof of full-density structural production [36]. The H13 comparison lists BJT apparent throughput of 5.5 kg/h, but does not establish equivalent accepted-part throughput across printing, sintering and inspection [13]. Section 6.5 therefore compares complete chains rather than using printer speed as an economic proxy.

### 3.4. Metal Material Extrusion

Metal MEX extrudes a metal-filled binder feedstock and uses debinding and sintering to obtain a metallic component [37,38,39]. 316 L and 17-4PH are established research or commercial systems; selected tool steels and nickel or copper grades are also possible, but availability does not establish automotive qualification. A Metal X specification gives a 300 × 220 × 180 mm printer envelope [40]. Furnace capacity, the green-to-sintered scale factor, supports and debinding constraints further reduce usable component size.

The dimensional challenge is anisotropic shrinkage and distortion, not simply nozzle positioning. Sintering shrinkage in metal extrusion is commonly of the order of 15–20% in linear dimensions, varying with feedstock and process [37,39]. This is compensated by oversizing; it is not an accuracy tolerance. Thick-to-thin transitions, unsupported spans and differences in local packing can cause nonuniform deformation. Machining allowance and sintering supports should therefore be designed into critical datums.

Inter-road voids and incomplete densification can survive the thermal cycle, as demonstrated in 316 L research [41]. In the H13 comparison, the two extrusion-and-sintering systems reached relative densities of 90.7% and 94.0% [13]. Those values describe particular implementations and must not be generalised to every MEX alloy or machine. They nevertheless show why density, tensile properties and heat-treatment response cannot be transferred from LPBF to a sintered part. Selected auxiliaries may tolerate these limitations, whereas safety-relevant parts require independently demonstrated properties.

## 4. Automotive Relevance of Metal AM Technologies

The automotive relevance of a process follows from the requirements of a qualified part and its manufacturing chain, not from a single maturity score assigned to a technology family.

LPBF is a candidate when internal channels, geometric integration or mass reduction compensate for supports, powder handling, post-processing and inspection [19,42]. Casting, forging or machining remain necessary comparators: an AM-shaped part may offer a different function, so both a like-for-like replacement and a functionally redesigned alternative should be considered.

BJT is a candidate for batches of smaller parts where avoiding dedicated mould tooling is valuable, but it must be compared with metal injection moulding (MIM), investment casting and machining. Volkswagen’s industrialisation statements establish interest and development activity [7]; they do not demonstrate a universal cost or production-volume threshold. Final density, shrinkage yield and furnace utilisation can overturn an apparent printer-throughput advantage.

DED is a candidate for local repair, feature addition and larger preforms when machining access is available. Laser-deposition repair evidence is discussed in [30], while the hybrid-manufacturing rationale is developed in [9]. WAAM belongs within DED and offers a deposition-rate advantage for suitable larger geometries [28]. None of these sources establishes that arbitrary automotive chassis or suspension parts are economical to repair.

MEX may be sufficient for selected small auxiliaries where dimensional requirements, strength and annual demand are compatible with debinding and sintering [37,41]. A metal process is unnecessary if a polymer fixture already meets stiffness, temperature and wear requirements. Technology selection should first exclude infeasible routes rather than reward a process for being additive.

Table 2 compares MAM process chains. LPBF is particularly suitable for internal channels and highly integrated geometries, although supports, stress relief, and finishing may be required. Binder jetting is a potential option for repeated production of smaller parts, but its finished-part output depends strongly on furnace capacity, batch utilisation, green-part handling, and shrinkage compensation. Laser-based DED is well-suited to local repair, feature addition, and larger preforms, with machine access representing a key feasibility constraint. WAAM offers high deposition rates for large preforms and selected structural applications, but these rates should not be interpreted as finished-part productivity because machining and thermal management are generally required. Metal material extrusion can be used for selected smaller functional components and auxiliary parts, although debinding, furnace constraints, anisotropic shrinkage, and finishing reduce the usable sintered-part envelope relative to the nominal printer size. Overall, process selection for automotive applications must consider the complete manufacturing chain rather than relying only on machine envelope or deposition rate.

The values above illustrate particular equipment or studies. They are not directly normalised across alloys or printing and sintering routes. Relative density is addressed in Section 5. Minimum feature size, dimensional tolerance and surface roughness require a selected machine, orientation, alloy and finishing state; the evidence does not justify a universal numerical range for every category.

### Requirement-Led Selection and Conventional Alternatives

Gate 1—Define function and baseline. Specify loading spectrum, temperature, corrosion or conductivity duty, life, inspection needs, production quantity and delivery constraints. Compare against casting, forging, sheet forming, fabrication or machining with the same required function. Sheet forming remains a strong baseline for thin, high-volume panels; forging for heavily loaded conventional geometries; casting for repeatable complex volumes; and machining for accessible features without a need for dedicated tooling.

Gate 2—Select feasible alloy–process–machine combinations. Reject routes whose qualified envelope, heat treatment, support removal, internal cleaning, shrinkage or machining access cannot meet the drawing. For a cooled steel insert, LPBF and a machined tool base may be viable; for an accessible worn die surface, DED plus machining may be viable; for a small 316 L bracket, compare BJT, MEX, machining and MIM before selecting equipment.

Gate 3—Establish qualification evidence. Require representative material and component tests, traceable feedstock, a controlled processing window and an inspection plan. Fatigue-critical designs need surface- and defect-sensitive evidence in the final condition. No route passes this gate solely because average density or tensile strength is high.

Gate 4—Compare accepted-part economics and deployment risk. Assess the complete accepted-part cost, considering yield, batch utilisation, post-processing, qualification and downtime. Retain the conventional route when the functional benefit or total-cost saving is not demonstrated. This ordered screening is a proposed decision framework rather than a numerical model validated against plant-selection outcomes.

## 5. Metallic Materials and Property Considerations

Alloy selection must begin with the component’s duty: static and cyclic loading, temperature, corrosion, wear, conductivity and dimensional stability. The process, machine, feedstock and post-processing route must then be qualified together. Table 3 separates the functional driver from example components and links the material issues to relevant sources.

A recurring issue in AM is that the same nominal alloy can behave very differently depending on the process route. Rapid solidification, cyclic reheating, anisotropic thermal gradients, porosity formation and residual stress can produce microstructures that diverge significantly from cast or wrought references [17,18,19,22,23,72,73,74,75]. Accordingly, the engineering question is not simply whether a given alloy is printable, but whether the printed microstructure, defects and post-processed state satisfy the intended service requirements.

The metallurgy of metal AM is intrinsically linked to repeated rapid heating, solidification and reheating. These conditions can generate directional grain structures, local heterogeneity and anisotropy that are not captured by nominal alloy composition alone [76,77]. More broadly, reviews of AM methods and materials emphasise that feedstock characteristics, thermal history, defects and process parameters must be treated as an integrated process–structure–property chain when transferring a material from conventional manufacture to AM [78,79,80,81].

### 5.1. Aluminium Alloys

Aluminium alloys are central to automotive lightweighting and electrification. Among currently printable alloys, AlSi10Mg remains the best established in LPBF because of its relatively good processability and fine as-built microstructure. It is widely used in demonstrators, housings, thermal-management parts and lightweight brackets. However, its fatigue behaviour remains strongly dependent on porosity, surface condition and post-processing. Heat treatment can reduce residual stress but may also coarsen microstructure and alter strength-ductility balance [23,43,44,73,74,75,82,83].

Experimental work on LPBF AlSi10Mg further shows that microstructure, high-cycle fatigue and fracture behaviour are closely connected to the thermal history and solidification conditions of the process [84]. This evidence supports treating fatigue qualification as a process-specific requirement rather than inferring cyclic performance from static strength alone.

Scandium- and zirconium-containing aluminium alloys can exploit rapid-solidification microstructures, but powder cost and supply remain relevant to automotive selection [45]. Grain-refining approaches also extend the range of high-strength aluminium alloys that can be processed by LPBF [85]. For aluminium WAAM, oxide contamination, hydrogen-related porosity and heat accumulation require controlled feedstock, shielding and interpass conditions [86,87]. These challenges are alloy- and process-specific rather than proof that all aluminium deposition is harder than all steel deposition.

For aluminium alloys, corrosion performance must be considered together with strength and fatigue. AM-specific microstructures, porosity and surface condition can modify localised corrosion behaviour relative to conventionally processed material, and the response is sensitive to alloy chemistry and post-processing [11,46,47]. This is particularly relevant for automotive parts exposed to humidity, road salts or thermal cycling, where a mechanically acceptable as-built state may still require surface finishing, heat treatment or protective strategies.

### 5.2. Steels

Firstly, 316 L is used in LPBF, BJT, MEX and DED. Its applications depend on corrosion resistance and adequate ductility, while lack of fusion, porosity, anisotropy and residual stress still require control [41,48,49,50,51,87]. Compared with cast or powder-HIP (hot isostatic pressing) 316 L, LPBF can produce higher yield strength through its finer microstructure, but subsequent HIP or heat treatment changes the comparison [51]. This cannot be translated directly into a fatigue advantage.

For tooling, H13 hot-work steel and precipitation-hardening maraging steels serve different functions and should not be grouped solely as printable steels. H13 requires control of cracking, preheating, retained phases and tempering; the desired combination is hot strength, toughness and thermal-fatigue resistance rather than maximum as-built hardness [52,53]. 17-4PH requires control of composition, phase balance and ageing condition. Tooling qualification also includes wear, thermal cycling and cooling-channel integrity. The H13 multi-process comparison shows why high average density alone does not establish uniform microstructure or interchangeable heat-treatment response [13].

### 5.3. Titanium Alloys

Titanium alloys, especially Ti-6Al-4V, offer high specific strength and excellent corrosion resistance, but their automotive use is generally restricted to high-performance, racing or prestige-vehicle components because of raw material cost and machining economics. AM improves the business case for titanium only when the design benefits are substantial. The Bugatti titanium brake calliper illustrates this logic: the value lies in extreme weight-performance positioning, technical demonstrability and geometric freedom, not in routine mass-market economics [1,3,4].

For Ti-6Al-4V, build orientation and thermal history affect microstructure and tensile response [54,55,56,57]. LPBF and EB-PBF produce different material states [58], and wire-fed laser deposition is another distinct route [59]. A qualified LPBF material specification therefore cannot, without additional evidence, serve as qualification of a DED or EB-PBF component. Heat treatment and HIP must be included in any property comparison.

Fatigue and damage tolerance are especially important for titanium components intended for cyclic automotive loading. Experimental studies have shown that LPBF Ti-6Al-4V fatigue performance depends strongly on defects, surface condition and microstructure [60,61,62], while crack-propagation behaviour provides an additional criterion beyond conventional tensile testing [63]. AM also enables cellular Ti-6Al-4V architectures, but their mechanical response depends on lattice geometry and cell size, so lightweight design requires structural validation at both material and architecture levels [88,89].

### 5.4. Nickel Alloys and Copper Alloys

Nickel alloys such as Inconel 625 and 718 can support selected exhaust, thermally loaded prototype or specialised tooling functions when their cost is justified. Much of the cited experimental evidence concerns general high-temperature engineering, including aerospace propulsion; it is transferable metallurgy rather than evidence of automotive series production [64,65,66,67,68,90]. Copper and copper alloys instead address electrical and thermal conductivity, with suitability depending on density, composition and the surface condition of internal channels [69,70,71].

Pure copper is difficult to process with conventional near-infrared lasers because of energy-coupling and heat-flow constraints [69,70,71]. Green and blue laser sources improve absorption at shorter wavelengths; commercial green-laser LPBF and experimental blue-laser DED should be distinguished [14,15,21]. A blue-laser DED study used 445 nm and examined porosity, dilution and building efficiency [15]. These benefits are particularly relevant to copper; they should not be transferred automatically to nickel alloys, for which alloy-specific solidification and cracking behaviour remain central.

### 5.5. Condition-Specific Benchmarking and Qualification

For automotive deployment, the most relevant property domains are usually tensile behaviour, fatigue resistance, dimensional stability, thermal performance, wear, corrosion and reproducibility. Static tensile strength alone is rarely decisive. Fatigue, in particular, is often the limiting property because it is highly sensitive to process defects, surface roughness and residual stress [17,18,19,22,23,72,73,74,75]. This is why post-processing—machining, shot peening, heat treatment or HIP when justified—remains central to the acceptance of critical AM metal parts [91,92,93,94,95,96,97,98,99,100,101,102,103,104]. Table 4 summarizes the condition-specific mechanical benchmarks and the limitations of comparisons across manufacturing routes.

Another decisive issue is process-to-process consistency. A component that performs well in a single build is not necessarily viable for industrialisation. The automotive sector, with its strong cost pressure and quality discipline, requires stable digital process chains, repeatable feedstocks, controlled post-processing and data-driven qualification strategies [105,106]. This is especially relevant for binder jetting and MEX, where final properties emerge after sintering rather than only during printing [72,96,107,108,109].

**Table 4:** Condition-specific mechanical benchmarks and limits of cross-route comparison.

Material and Route	Condition	Quantitative Result	Comparison Limit
AlSi10Mg LPBF [110]	Polished fatigue surface, Ra < 0.8 μm; R = −1; 10 Hz	UTS 435.2 MPa; thermographic fatigue-limit estimate 147.5 MPa	Same study as the cast row. Estimate is not a design allowable or a fitted 10^7^-cycle endurance strength.
AlSi10Mg gravity cast [110]	Polished fatigue surface, Ra < 0.8 μm; R = −1; 10 Hz	UTS 328.3 MPa; thermographic fatigue-limit estimate 183.2 MPa	Higher LPBF tensile strength did not imply higher estimated fatigue resistance.
AlSi10Mg LPBF vertical/horizontal; cast [16]	No heat treatment; ground surfaces; axial cyclic tests	Basquin fit (σf′ MPa, b): (553, −0.1442); (651, −0.1449); (488, −0.1028)	Source regression coefficients, not interchangeable fatigue limits; no extrapolation is made here.
316L LPBF; LPBF + HIP; powder HIP; cast [51]	Route-specific microstructure and thermal history	Direct comparative study; LPBF has higher yield strength in its investigated state	Matched fatigue allowables are not extracted from this source; no universal numerical fatigue ranking is assigned.
Ti-6Al-4V LPBF/EB-PBF [58,100,102]	Route, HIP and surface state must be distinguished	Comparative AM and post-processing evidence; not a matched cast–forged–LPBF dataset	No complete identical-condition cast/forged/AM property grid established in this review.

UTS denotes ultimate tensile strength; Ra is arithmetic mean profile roughness; R is minimum divided by maximum cyclic stress. The AlSi10Mg thermographic values were inferred using the method of the cited study and should not be equated to conventional statistical fatigue allowables. The cast and AM materials have separate processing histories. No heat-treatment equivalence is assumed where not established by the source.

A complete alloy-by-alloy table of cast, forged and AM tensile and fatigue limits under identical conditions cannot be supported by the available comparisons. AlSi10Mg is primarily a casting alloy; substituting a forged wrought alloy of different chemistry would confound material and process effects. For steel and titanium, matched chemistry, heat treatment, orientation, surface state, stress ratio, temperature, cycle count and survival probability must be controlled. The absence of a matched benchmark is an evidence gap, not proof of inferiority or superiority.

## 6. Applications in Automotive Components and Auxiliary Elements

Direct vehicle parts and auxiliary manufacturing applications have different qualification burdens and economic baselines. Table 5 summarises the reported application classes, materials, process routes and evidence of deployment. Production volume is stated only when the cited source reports a usable figure; a missing value is not converted into an assumed production class.

### 6.1. Direct Vehicle Components

**Figure 5:**
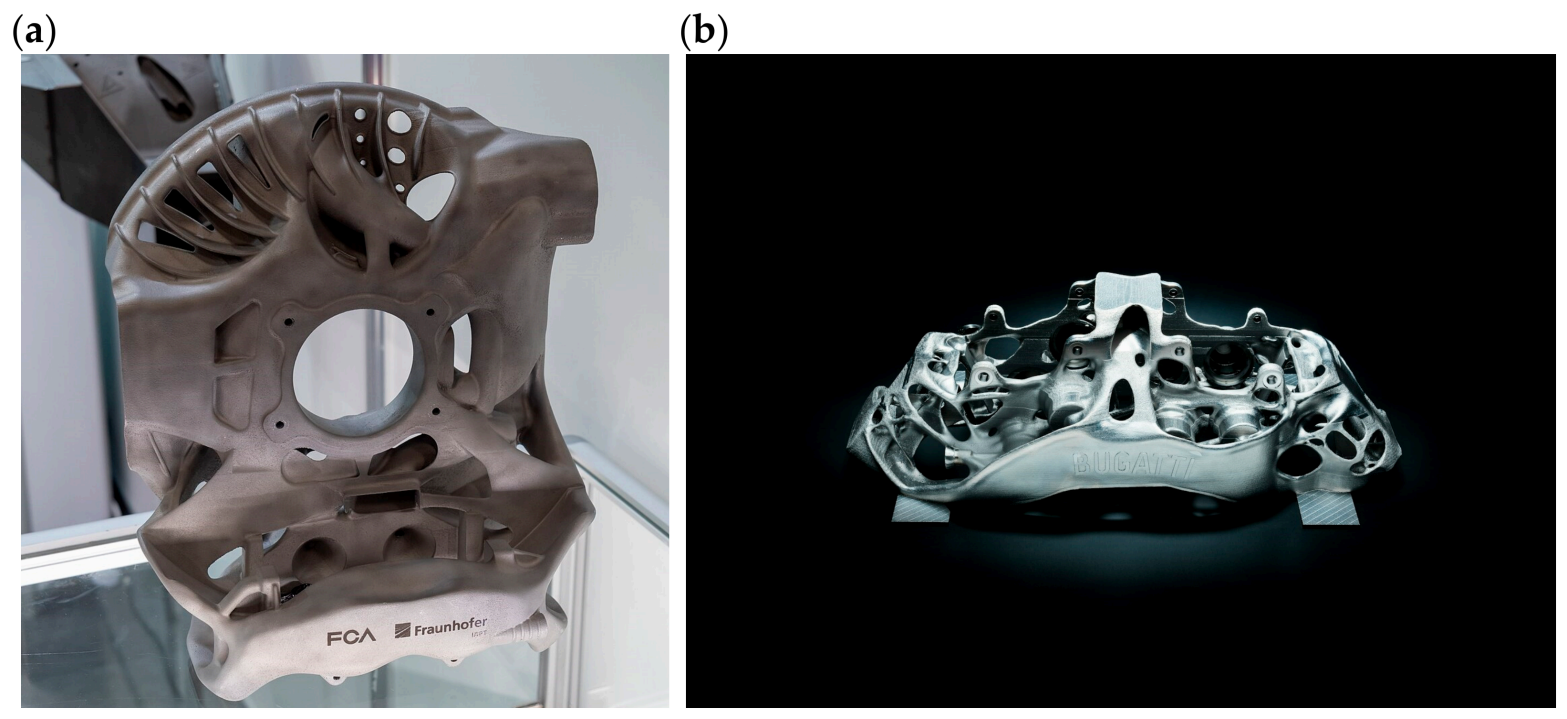
Automotive metal additive manufacturing demonstrators. (**a**) Topology-optimised wheel carrier with integrated brake-calliper functions, jointly developed by Fiat Chrysler Automobiles and Fraunhofer IAPT for a sports-car application. Image credit: Matti Blume, “Professional MotorSport World Expo 2024, Cologne (P1031266)”, Wikimedia Commons, licenced under CC-BY-SA 2.0+ [116]. (**b**) LPBF-fabricated eight-piston monobloc Bugatti brake-calliper demonstrator in Ti–6Al–4V, developed in cooperation with Laser Zentrum Nord, now Fraunhofer IAPT [20]. Image credit: Roie Galitz/Fraunhofer IAPT, “Bremssattel Bugatti”, Wikimedia Commons, licenced under CC BY-SA 4.0 (Both images were cropped and resized for layout) [117].

Porsche reports aluminium LPBF pistons developed for the 911 GT2 RS with a 10% mass reduction relative to the forged production pistons and an integrated cooling passage [6]. This comparison reflects a component-specific redesign and should not be interpreted as evidence that LPBF aluminium intrinsically outperforms forged material. BMW has also documented an aluminium roof-mechanism bracket for the i8 Roadster as a series application [111]. At the subsystem level, Fraunhofer IAPT and Fiat Chrysler Automobiles (FCA) developed a topology-optimised, additively manufactured wheel carrier with integrated brake-calliper functions for a sports-car application. The one-piece bionic prototype consolidated 12 conventionally manufactured parts and was reported to achieve a 36% mass reduction while substantially simplifying assembly [115]. Nevertheless, it should be regarded as a development prototype rather than a demonstrated series-production component. Bugatti’s eight-piston monobloc brake-calliper demonstrator was produced from Ti–6Al–4V by LPBF—described by the manufacturer as selective laser melting—and weighed 2.9 kg, compared with 4.9 kg for the forged-aluminium production calliper. Its manufacture required a 45 h build, followed by heat treatment, support removal, surface finishing, and 11 h of five-axis machining [20]. Figure 5 illustrates these two automotive demonstrators, highlighting part consolidation and topology-driven lightweighting in the FCA–Fraunhofer suspension component and material- and process-enabled mass reduction in the Bugatti brake calliper.

**Figure 6:**
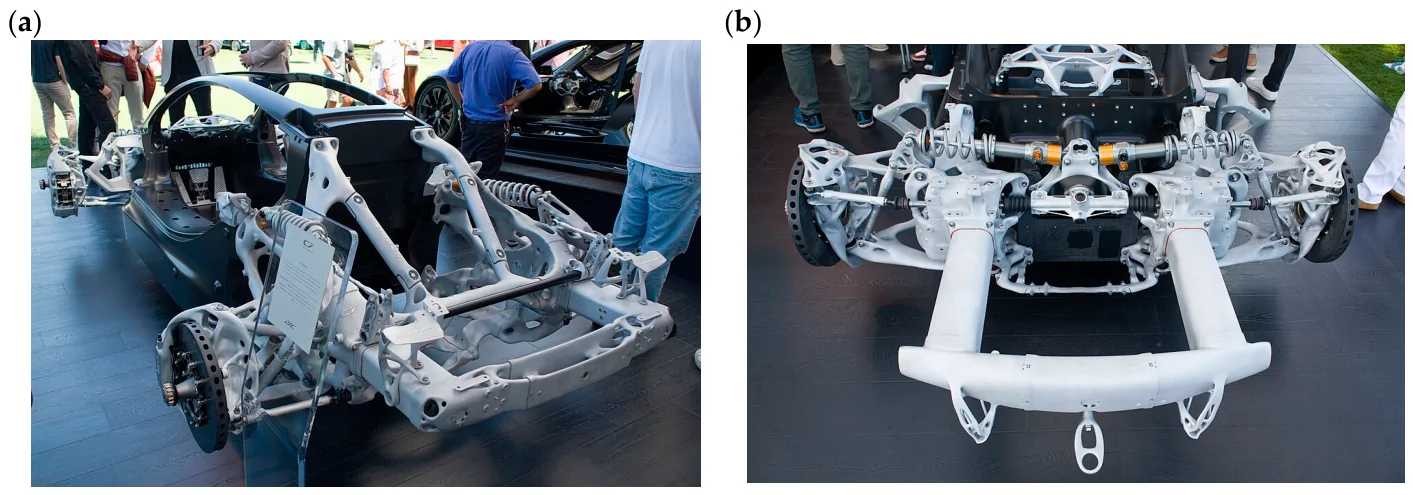
Czinger 21C chassis displayed at The Quail 2023: (**a**) front view [119] and (**b**) rear view [120]. The vehicle structure is a multi-component assembly associated with the LPBF-based Divergent manufacturing route and should not be interpreted as a monolithic DED build. Photographs by Prova MO, reproduced from Wikimedia Commons under the CC BY-SA 4.0 licence.

Large assemblies do not imply that the entire structure was printed by DED. Figure 6 presents front and rear views of the Czinger 21C chassis, which is assembled from individually manufactured and joined components. The source describes LPBF within the Divergent manufacturing route [112]. Its system-level integration must therefore be distinguished from a monolithic DED build. BMW separately reports the development of WAAM for larger structural components and a prospective series application from 2027 [5,118]. That date is a company-stated target, not demonstrated serial output as of September 2026.

**Figure 7:**
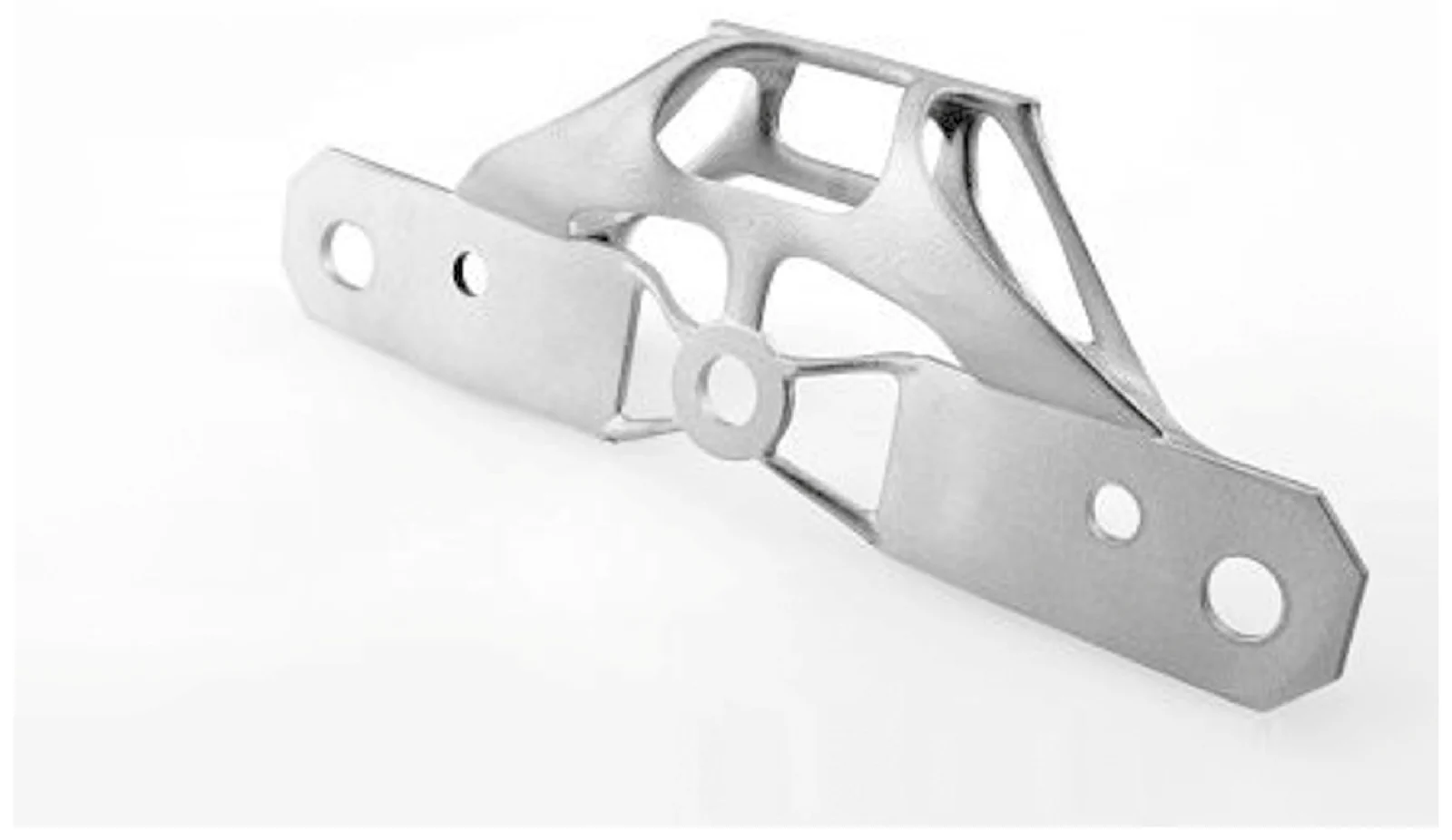
Topology-optimised automotive bracket redesigned for metal Binder Jetting, with a reported mass reduction of 47% compared with the conventional design [122]. Image courtesy of The ExOne Company; reproduced with permission.

Another automotive application is the topology-optimised bracket shown in Figure 7. According to the published case description, the component was redesigned for metal Binder Jetting by a major automotive manufacturer, resulting in a reported mass reduction of 47% compared with the conventional design while also reducing the number of welded joints required for assembly [121]. This example illustrates the potential of design for additive manufacturing to reduce component mass and assembly requirements. Nevertheless, process selection must still account for mechanical and thermal loads, dimensional tolerances, accessible finishing surfaces, and inspection requirements. For electric drives, compact cooling passages and conductive copper features are plausible opportunities, supported by copper-process developments [14,15,21]; laboratory manufacturability should not be described as vehicle-level validation.

### 6.2. Tooling, Jigs, Fixtures and Assembly Aids

Factory production aids should be screened first for whether metal is necessary. Polymer grippers, ergonomic handling aids and lightweight positioning fixtures must not be counted as evidence of MAM deployment. Metallic candidates include wear-resistant contact inserts, hot-work tooling, welding-contact hardware and stiffness-critical fixture elements. A hybrid fixture can combine a conventional frame, replaceable printed metal inserts and polymer handling surfaces; only the metal portions fall within the scope of this review [9,42].

The economic comparator for a customised fixture is a machined or fabricated tool with equivalent life, accuracy and delivery time. Faster availability may reduce line downtime, but this benefit requires a documented counterfactual and cannot be inferred from printing time alone. Conformal-cooling examples are discussed in Section 6.3 to avoid counting the same application twice.

### 6.3. Moulds, Dies and Conformal Cooling

**Figure 8:**
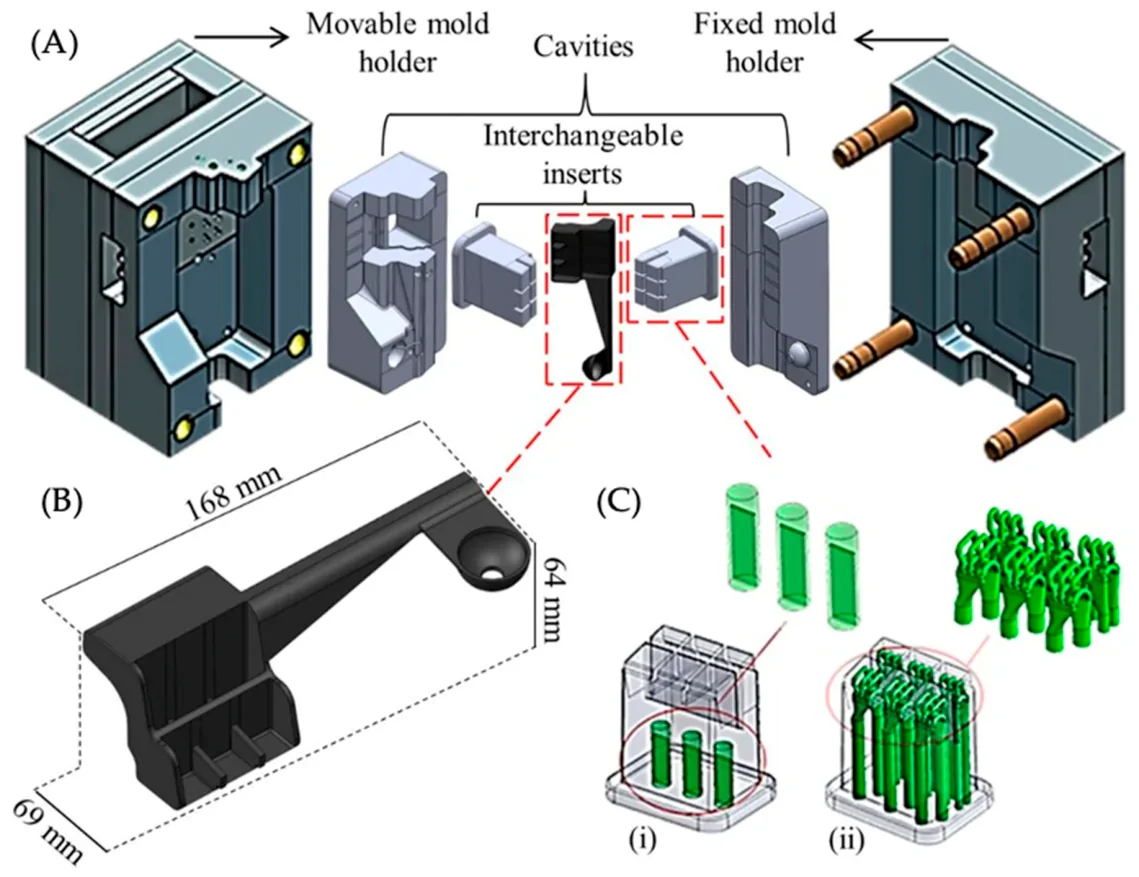
Experimental injection-mould configuration and interchangeable cooling inserts for an automotive polypropylene component: (**A**) mould assembly and interchangeable inserts; (**B**) automotive workpiece geometry; and (**C**) conventional machined baffle cooling channels (i) and LPBF-manufactured conformal cooling channels (ii). Reproduced without modification from Popiolek et al. [123], © 2026 the authors, under the Creative Commons Attribution 4.0 International licence © CC BY 4.0); no changes were made.

LPBF allows cooling passages to follow local thermal demand in steel inserts, while final contact faces and datums can be machined. Audi and EOS report 12 AM segments in four hot-forming tools used to manufacture body panels, including for the Audi A4 [113]. Popiolek et al. experimentally compared two sets of interchangeable mould inserts used to manufacture the same automotive polypropylene component: a conventionally machined PH13-8Mo stainless-steel insert containing baffle cooling channels (BCCs) and a Corrax stainless-steel insert manufactured by LPBF with conformal cooling channels (CCC) [123]. Figure 8 shows the mould configuration, the automotive workpiece and the two cooling-channel arrangements. Under the reported experimental conditions, the minimum cooling time permitting acceptable automatic part extraction decreased from 23 s with the conventional insert to 17 s with the conformal-cooling insert, corresponding to a reduction of approximately 26%. The parts manufactured using the CCC insert were also reported to be 83% more dimensionally precise than those produced using the conventional insert. No statistically significant differences were identified in the tensile or impact responses, while the evaluated crystallinity and viscoelastic behaviour did not indicate detrimental changes attributable to the shorter cooling cycle [123]. These results are specific to the investigated component, polypropylene grade, insert materials and processing conditions and should not be interpreted as general performance or payback estimates.

### 6.4. Repair, Remanufacturing and Spare Parts

Repair is attractive only if restoring a retained asset is preferable to replacement. A hybrid repair chain comprises damage assessment and scanning, removal of degraded material, substrate and interface preparation, deposition, appropriate heat treatment, machining and inspection [9,30]. For dies and moulds, qualification must address dilution, local hardness, heat-affected-zone cracking and thermal fatigue. Depositing fresh material does not remove damage elsewhere in the retained component. Repair of low-cost frames, chassis or suspension parts is therefore not assumed to be economical or acceptable; replacement may be the appropriate baseline.

Candidate repair assets are high-value tools or surfaces for which the retained geometry, replacement lead time and repairable damage support the business case. Spare-part manufacture is a separate activity: it requires verified geometry, material and inspection requirements, traceable digital revisions and a justified stock-versus-on-demand comparison. For WAAM, path planning and thermal history affect distortion [124,125,126,127], while interlayer rolling can modify grain structure and residual stress [128,129]. Recent reviews discuss unresolved quality and multi-material issues [87,130,131,132,133]. These results inform process development rather than establish interchangeable repairs for automotive safety-critical parts.

### 6.5. Economics and the Accepted-Part Cost Baseline

The economic viability of MAM should be assessed across the complete manufacturing chain. Printing represents only one part of the cost of delivering an accepted component. Feedstock, design preparation, equipment utilisation, post-processing, inspection and rejected parts can substantially influence the final cost. Comparisons with conventional manufacturing should therefore consider components that meet equivalent functional and quality requirements.

The balance between initial investment and recurring production costs is particularly relevant when comparing binder jetting (BJT) with metal injection moulding (MIM). BJT can reduce the need for dedicated mould tooling, making it attractive for development programmes, design changes and smaller production batches. MIM can distribute its tooling investment across larger production volumes. However, the economic balance also depends on component geometry, material, furnace utilisation, finishing requirements and acceptance rates. Production volume alone is therefore insufficient to establish a general preference for either route.

For tooling applications, the value of additive manufacturing may extend beyond the purchase price of the tool. Conformal cooling can improve thermal management and shorten moulding cycles, potentially offsetting higher manufacturing and qualification costs. The reported armrest-mould case illustrates this opportunity [114]. Nevertheless, the economic benefit depends on production demand, tool life, maintenance requirements and whether the released machine capacity can be used productively. A shorter cycle does not automatically translate into equivalent financial savings.

Repair and remanufacturing require a similar assessment over the remaining service life of the component. Restoring a high-value part may be attractive when it avoids replacement costs or long delivery times, provided that inspection and qualification establish adequate performance. The comparison must also account for preparation, deposition, finishing, downtime and the expected life of the repaired part. For spare parts, on-demand manufacturing may reduce inventory and improve availability, but these benefits must be weighed against data preparation, qualification and uncertain demand.

Several studies have assessed the economic competitiveness of MAM against conventional manufacturing technologies. In one study [134], a 17-4 PH stainless steel trigger for a hydraulic cylinder housing was manufactured using MEX and compared with CNC machining. Under optimised design and production conditions, MEX achieved a cost reduction of approximately 13.68% compared with CNC machining. However, debinding, sintering, and machining accounted for 49% of the total production cost and approximately 76% of the total production time. The study also highlighted the importance of optimising process parameters, component design, and equipment utilisation, as directly transferring the CNC-oriented design to MEX increased production time by 158% and production cost by 27%. Another study [135] evaluated the technical, environmental, and economic trade-offs of LPBF compared with conventional machining for a representative metal component in the aerospace sector, showing that its economic competitiveness strongly depends on production volume and process conditions. In addition, a further study [136] compared the manufacturing costs of a high-strength low-alloy steel component produced by WAAM, LPBF, and CNC machining, showing that WAAM can be a cost-effective option for components of medium complexity, depending on the production volume. Although these components are not directly related to the automotive sector, these studies provide relevant examples of the factors governing the economic viability of MAM, including component design, production volume, process selection, equipment utilisation, and post-processing requirements.

Economic selection should consequently be based on the specific component and production context. MAM is most attractive when its capabilities—such as functional integration, reduced tooling requirements, shorter lead times or extended asset life—provide benefits that justify the complete cost of manufacturing and qualification. These advantages should be demonstrated for the intended application rather than inferred from printing speed or material savings alone.

## 7. Future Lines for Research and Industrial Adoption

Future adoption depends on closing application-specific qualification and economic gaps. Table 6 separates available capabilities from prospective directions. The indicative horizons are planning assumptions made in this synthesis, except where a company announcement is explicitly identified; they are not forecasts derived from a longitudinal dataset.

Design for additive manufacturing (DfAM) should connect function to a manufacturable and inspectable chain [42,137,138]. LPBF design requires powder-exit paths, support access, channel cleanability, distortion compensation and machining datums. BJT and MEX require allowance for green strength, debinding access, shrinkage and sintering supports. DED design requires substrate access, bead-scale features and machining stock. A topology-optimised part that cannot be depowdered, inspected or repeatedly manufactured is not a qualified design.

ISO/ASTM 52920:2023 describes AM production-site and process-quality requirements that supplement a quality-management system, including an automotive system such as International Automotive Task Force (IATF) 16949 [139]. This does not constitute automatic component approval. A deployment plan must define material and process specifications, feedstock and machine traceability, representative coupon testing, dimensional inspection, defect acceptance, fatigue evidence and change control. Supplier production approval and customer-specific requirements apply according to the programme; no single AM certificate substitutes for them.

Monitoring and artificial intelligence (AI) can support anomaly detection, but their ability to predict acceptance must be validated against independent inspection and mechanical evidence [107,108,109,140]. Important barriers include transfer between machines and alloys, data imbalance, undetected defect classes and false acceptance. The necessary research result is a demonstrated reduction in decision error on unseen builds, not merely a high training accuracy. Full replacement of final inspection is not assumed.

Hybrid chains are already technically feasible, but industrial expansion requires better control of interface properties, machining accessibility and repeatability [9,30]. For repair, acceptance should compare restored life and total downtime against replacement; for new parts, it should compare functional integration and accepted-part cost against casting, forging, fabrication or machining.

Sustainability should be evaluated with an explicit functional unit, system boundary and service life [141,142,143,144,145,146]. The NIST characterisation framework provides a basis for measuring process inputs and outputs [146]. Life-cycle comparisons must include feedstock production, build utilisation, electricity, supports, furnace cycles, machining, rejects, repair life and transport. A suitable functional unit may be one accepted tool delivering a specified number of cycles or one component delivering a specified vehicle service life. Lower material waste alone is insufficient evidence of lower environmental impact.

Electrification motivates research on cooling and conductive components, but it also changes the incumbent design and manufacturing baseline [14,15,21]. Copper-channel processability is not equivalent to demonstrated pressure tightness, coolant compatibility or electrical reliability in a vehicle. Priorities include conductivity after heat treatment, corrosion at material interfaces, internal-channel roughness and repeatability under thermal cycling.

**Table 6:** Research and deployment priorities with conditional planning horizons.

Direction	Evidence or Present State	Main Barrier and Required Evidence	Indicative Horizon
DfAM and hybrid finishing	Methods and selected industrial chains available [9,42]	Demonstrate inspection access, repeatability and total cost in the final design	Near term, 0–2 years for selected qualified cases; synthesis assumption
BJT/MEX industrialisation	Commercial systems; heterogeneous sintered properties [13,33,37]	Map shrinkage, furnace utilisation, local defects and component fatigue	2–5 years for broader selected use; no universal serial-production forecast
Monitoring and AI	Process sensing and prediction research [107,108,109,140]	Independent-build validation, false acceptance and transfer between machines	2–5 years for validated decision support; replacing inspection remains uncertain
WAAM structural deployment	BMW reports future series use from 2027 [118]	Component fatigue, distortion, interfaces and production control	2027 is a company target; achievement not inferred
Repair and circularity	Deposition/machining chains demonstrated [9,30]	Restored-life evidence and full lifecycle comparison against replacement	Application-specific; no single maturity date
Copper and electric-drive functions	Green LPBF available; blue DED under study [14,15,21]	Leak tightness, conductivity, coolant compatibility and thermal cycling	Selected deployments possible; broad vehicle qualification timing uncertain

## 8. Conclusions

MAM provides application-specific manufacturing options for automotive engineering. LPBF is suited to selected complex parts, BJT to potentially productive sintering-based batches, DED to local deposition and larger preforms, and MEX to selected smaller components. These roles are conditional on alloy, geometry, qualification and the entire post-processing chain, rather than a universal ranking of process maturity.

The available material evidence does not establish a general mechanical advantage over casting or forging. Condition-specific AlSi10Mg comparisons show that greater tensile strength can coexist with poorer fatigue performance. H13 comparisons show that process route strongly affects densification. Design values must therefore come from the qualified material state and service-relevant testing rather than nominal alloy or average density alone.

Industrial examples support further use of MAM in tooling, selected direct components and lifecycle support, but they do not establish general profitability. The proposed selection framework combines functional need, process feasibility, qualification and cost per accepted part. The economic assessment highlights why annual demand, yield, furnace utilisation and avoided downtime should be considered when evaluating the economic suitability of MAM. Incomplete search records and heterogeneous reporting limit the strength of population-wide conclusions. Further progress requires traceable comparative datasets and application-specific economic validation.

## Figures and Tables

**Table 1:** Scope comparison and positioning of the present synthesis. “Emphasis” describes the main scope, not total absence of other subjects.

Contribution	Principal Emphasis	Relationship to This Review
Metal automotive review [1]	Vehicle parts and components	Closest scope; used as an automotive baseline
Automotive mini review [2]	AM processes and materials including nonmetals	Broader material scope; metal-only decisions examined here
Automotive conference review [3]	Applications, benefits and limitations	Related sector overview
Automotive status review [8]	Status, challenges and future perspectives	Automotive status and implementation context
Hybrid-manufacturing study [9]	Industrial constraints and hybrid solutions	Basis for combining deposition with conventional operations
Process/materials reviews [10,11]	Wire-arc processing/corrosion of AM aluminium	Depth in individual process and property domains
Present review	Metal process–alloy–application decisions	Explicit evidence categories, qualified-chain selection and conventional cost baseline

**Table 2:** Process-chain comparison using documented examples rather than universal capability limits.

Route	Documented Numerical Example	Materials and Chain Constraints	Automotive Interpretation
LPBF	TruPrint green: Ø300 × 400 mm; 30–150 μm layers; 8–100 cm^3^/h [21]	Example platform for Cu/Cu alloys. Other platforms: Al, steels, Ti, Ni [19]. Supports, stress relief and finishing as required.	Internal channels and integrated geometries; machine values are not accuracy limits.
BJT	HP S100 green envelope 430 × 309 × 170 mm [33]; H13 study: 5.5 kg/h apparent throughput [13]	316 L/17-4PH commercial examples. Green handling, sintering supports, shrinkage compensation and finishing.	Candidate for repeated smaller parts. Furnace yield and utilisation govern finished output.
DED-LB	H13 wire-laser study: 1–2 kg/h [13]; envelope is cell-specific.	Steels, Ni and Ti; selected Al/Cu. Bond quality, shielding, dilution and machining stock.	Local repair, feature addition and larger preforms; machine access is a feasibility gate.
WAAM within DED	Approximately 1–10 kg/h in the wire-arc literature [28].	Wire alloys; interpass temperature, residual stress and bead-scale geometry. Usually requires machining.	Larger preforms and selected structural development; rate is not finished-part output.
Metal MEX	Metal X printer envelope 300 × 220 × 180 mm [40]; linear sintering shrinkage often 15–20% [37,39].	316 L/17-4PH and selected other alloys. Debinding, furnace constraints, anisotropic shrinkage and finishing.	Selected smaller functional parts or auxiliaries; printer size exceeds usable sintered-part envelope.

**Table 3:** Material drivers, automotive examples and processing constraints.

Family and Alloys	Functional Driver	Component Examples	Issues and Candidate Routes
Aluminium: AlSi10Mg; Al–Mg–Sc–Zr	Low mass; thermal performance	Pistons, brackets, housings	LPBF; selected DED. Defects, heat-treatment response, corrosion and fatigue [11,43,44,45,46,47].
Steels: 316 L; 17-4PH; H13	Corrosion resistance; strength; wear and hot-work life	Fixtures, tool inserts, spare parts	LPBF, BJT, MEX, DED; grade-specific phase balance, cracking and heat treatment [13,41,48,49,50,51,52,53].
Titanium: Ti-6Al-4V	Specific strength; corrosion resistance	Performance brackets; calliper demonstrator	LPBF, EB-PBF, DED. Cost, surface-sensitive fatigue and thermal history [54,55,56,57,58,59,60,61,62,63].
Nickel: Inconel 625/718	High-temperature capability	Selected hot components or tooling	LPBF, DED. Segregation, heat treatment and application-specific cost [64,65,66,67,68].
Copper: pure Cu; CuCrZr	Electrical/thermal conductivity	Conductive features and cooling components	Selected LPBF/DED/BJT routes. Coupling, porosity and conductivity retention [14,15,35,69,70,71].

**Table 5:** Automotive application classes and the level of evidence reported.

Class and Example	Material and Process	Reported Scale or Status	Decision-Relevant Evidence
Direct part: Porsche piston [6]	Aluminium; LPBF	Advance-development project; annual output not reported	10% mass reduction vs. forged production piston; geometry changed.
Direct part: Bugatti calliper [20]	Ti-6Al-4V; LPBF	Demonstrator with planned vehicle trials	2.9 kg vs. 4.9 kg aluminium reference; 45 h print + 11 h milling.
Direct part: BMW roof bracket [111]	Aluminium; metal powder-bed process	i8 Roadster series application; quantity not resolved here	Named metallic component; aggregate AM counts are not component volumes.
Direct structure: Czinger [112]	Metal LPBF components in an assembly	2021 source describes an initial 80-vehicle programme	Programme size is not verified delivered output; assembly is not DED.
Factory aid: fixture contact insert	Steel; LPBF/MEX candidates	Application-dependent; no universal output figure	Select metal only where temperature, wear or stiffness justify it.
Tooling: Audi hot-forming segments [113]	Metal tool segments; LPBF	12 segments in four tools; reported production use	Conformal cooling integrated with conventional press-shop operations.
Tooling: armrest mould [114]	Tool core; EOS metal powder-bed route	Reported automotive moulding use	17% cycle reduction; maintenance interval increased in this case.
Repair/remanufacture [9,30]	Compatible substrate and deposit; DED + machining	Demonstrated repair principles; component-specific acceptance	Tool recovery may be viable; damage, restored life and replacement cost govern.
Spare parts	Specified alloy; qualified process	Demand-specific; no universal annual volume	Compare traceable on-demand supply against stockholding and conventional manufacture.

## Data Availability

No new data were created or analysed in this study. Data sharing is not applicable to this article.

## References

[1] Sarzyński B., Śnieżek L., Grzelak K. (2024). Metal Additive Manufacturing (MAM) Applications in Production of Vehicle Parts and Components—A Review. Metals.

[2] Yang J., Li B., Liu J., Tu Z., Wu X. (2024). Application of Additive Manufacturing in the Automobile Industry: A Mini Review. Processes.

[3] Mehdiyev Z., Felho C. (2023). Metal Additive Manufacturing in Automotive Industry: A Review of Applications, Advantages, and Limitations. Mater. Sci. Forum.

[4] Zhao N., Parthasarathy M., Patil S., Coates D., Myers K., Zhu H., Li W. (2023). Direct Additive Manufacturing of Metal Parts for Automotive Applications. J. Manuf. Syst..

[5] BMW Group Scalable, Automated, Open-Material: BMW Group Continues to Invest in Additive Manufacturing and Elevates the Technology to the Next Industrial Level. https://www.press.bmwgroup.com/global/article/attachment/T0455924EN/644774.

[6] Porsche AG Innovative Pistons from a 3D Printer for Increased Power and Efficiency. https://newsroom.porsche.com/en/2020/technology/porsche-cooperation-mahle-trumpf-pistons-3d-printer-power-efficiency-911-gt2-rs-21462.html.

[7] Volkswagen AG Volkswagen Plans to Use New 3D Printing Process in Vehicle Production in the Years Ahead. https://www.linkedin.com/posts/volkswagen-group_volkswagen-plans-to-use-new-3d-printing-process-activity-6811592642363723778-vtRu.

[8] Chen L., Ng N.P.H., Jung J., Moon S.K. (2023). Additive Manufacturing for Automotive Industry: Status, Challenges and Future Perspectives. Proceedings of the 2023 IEEE International Conference on Industrial Engineering and Engineering Management (IEEM).

[9] Stavropoulos P., Foteinopoulos P., Papacharalampopoulos A., Bikas H. (2018). Addressing the Challenges for the Industrial Application of Additive Manufacturing: Towards a Hybrid Solution. Int. J. Lightweight Mater. Manuf..

[10] Pattanayak S., Sahoo S.K. (2021). Gas Metal Arc Welding Based Additive Manufacturing—A Review. CIRP J. Manuf. Sci. Technol..

[11] Revilla R., Verkens D., Rubben T., De Graeve I. (2020). Corrosion and Corrosion Protection of Additively Manufactured Aluminium Alloys—A Critical Review. Materials.

[12] (2021). Additive Manufacturing—General Principles—Fundamentals and Vocabulary.

[13] Widomski P., Kaszuba M., Dobras D., Terefinko D., Kołodziński M. (2025). Comparative Study of Different Additive Manufacturing Methods for H13 Tool Steel. Materials.

[14] Khandpur M.S., Giubilini A., Iuliano L., Minetola P. (2024). On the Use of Green and Blue Laser Sources for Powder Bed Fusion: State of the Art Review for Additive Manufacturing of Copper and Its Alloys. Metals.

[15] Yamaguchi M., Kato N., Funada Y., Yachi T., Saikai A., Furumoto T. (2024). Influence of the Laser Power and Powder Feed Rate on the Porosity, Dilution, and Building Efficiency of Pure Copper Parts Fabricated via Directed Energy Deposition with a Blue Laser. Int. J. Adv. Manuf. Technol..

[16] Chmelko V., Šulko M., Škriniarová J., Margetin M., Gašparík M., Koščo T., Semeš M. (2023). Strength and Cyclic Properties of Additive vs. Conventionally Produced Material AlSi10Mg. Materials.

[17] DebRoy T., Wei H.L., Zuback J.S., Mukherjee T., Elmer J.W., Milewski J.O., Beese A.M., Wilson-Heid A., De A., Zhang W. (2018). Additive Manufacturing of Metallic Components—Process, Structure and Properties. Prog. Mater. Sci..

[18] Herzog D., Seyda V., Wycisk E., Emmelmann C. (2016). Additive Manufacturing of Metals. Acta Mater..

[19] Yap C.Y., Chua C.K., Dong Z.L., Liu Z.H., Zhang D.Q., Loh L.E., Sing S.L. (2015). Review of Selective Laser Melting: Materials and Applications. Appl. Phys. Rev..

[20] Bugatti Automobiles World Premiere: Brake Caliper from 3-D Printer. https://newsroom.bugatti.com/en/press-releases/world-premiere-brake-caliper-from-3-d-printer.

[21] TRUMPF TruPrint 5000 Green Edition. https://www.trumpf.com/filestorage/TRUMPF_Master/Products/Machines_and_Systems/02_Brochures/TRUMPF-TruPrint5000-Green-Edition-flyer-EN.pdf.

[22] King W.E., Barth H.D., Castillo V.M., Gallegos G.F., Gibbs J.W., Hahn D.E., Kamath C., Rubenchik A.M. (2014). Observation of Keyhole-Mode Laser Melting in Laser Powder-Bed Fusion Additive Manufacturing. J. Mater. Process. Technol..

[23] Aboulkhair N.T., Everitt N.M., Ashcroft I., Tuck C. (2014). Reducing Porosity in AlSi10Mg Parts Processed by Selective Laser Melting. Addit. Manuf..

[24] Yadroitsev I., Bertrand P., Smurov I. (2007). Parametric Analysis of the Selective Laser Melting Process. Appl. Surf. Sci..

[25] Kruth J., Mercelis P., Van Vaerenbergh J., Froyen L., Rombouts M. (2005). Binding Mechanisms in Selective Laser Sintering and Selective Laser Melting. Rapid Prototyp. J..

[26] Gu D.D., Meiners W., Wissenbach K., Poprawe R. (2012). Laser Additive Manufacturing of Metallic Components: Materials, Processes and Mechanisms. Int. Mater. Rev..

[27] Körner C. (2016). Additive Manufacturing of Metallic Components by Selective Electron Beam Melting—A Review. Int. Mater. Rev..

[28] Williams S.W., Martina F., Addison A.C., Ding J., Pardal G., Colegrove P. (2016). Wire + Arc Additive Manufacturing. Mater. Sci. Technol..

[29] Ding D., Pan Z., Cuiuri D., Li H. (2015). Wire-Feed Additive Manufacturing of Metal Components: Technologies, Developments and Future Interests. Int. J. Adv. Manuf. Technol..

[30] Graf B., Gumenyuk A., Rethmeier M. (2012). Laser Metal Deposition as Repair Technology for Stainless Steel and Titanium Alloys. Phys. Procedia.

[31] Mostafaei A., Elliott A.M., Barnes J.E., Li F., Tan W., Cramer C.L., Nandwana P., Chmielus M. (2021). Binder Jet 3D Printing—Process Parameters, Materials, Properties, Modeling, and Challenges. Prog. Mater. Sci..

[32] Ziaee M., Crane N.B. (2019). Binder Jetting: A Review of Process, Materials, and Methods. Addit. Manuf..

[33] HP Inc Revolutionize Metal 3D Printing with HP Scalable Platform Configurations. https://www.hp.com/gb-en/printers/3d-printers/products/metal-jet.html.

[34] Do T., Kwon P., Shin C.S. (2017). Process Development toward Full-Density Stainless Steel Parts with Binder Jetting Printing. Int. J. Mach. Tools Manuf..

[35] Bai Y., Williams C.B. (2015). An Exploration of Binder Jetting of Copper. Rapid Prototyp. J..

[36] Ramalingam M.S., Chaithanya Kumar K.N., Sharma S., Joshi S.S., Dahotre N.B. (2025). Binder Jet Additive Manufacturing: A Review of Modelling Approaches and Experimental Observations on Green Part Printing. Virtual Phys. Prototyp..

[37] Suwanpreecha C., Manonukul A. (2022). A Review on Material Extrusion Additive Manufacturing of Metal and How It Compares with Metal Injection Moulding. Metals.

[38] Nurhudan A.I., Supriadi S., Whulanza Y., Saragih A.S. (2021). Additive Manufacturing of Metallic Based on Extrusion Process: A Review. J. Manuf. Process..

[39] Gonzalez-Gutierrez J., Cano S., Schuschnigg S., Kukla C., Sapkota J., Holzer C. (2018). Additive Manufacturing of Metallic and Ceramic Components by the Material Extrusion of Highly-Filled Polymers: A Review and Future Perspectives. Materials.

[40] Markforged Metal X System Product Specifications. https://www-objects.markforged.com/craft/3d_printers_detail/metal-x/Metal_System_Datasheets.pdf.

[41] Damon J., Dietrich S., Gorantla S., Popp U., Okolo B., Schulze V. (2019). Process Porosity and Mechanical Performance of Fused Filament Fabricated 316L Stainless Steel. Rapid Prototyp. J..

[42] Thompson M.K., Moroni G., Vaneker T., Fadel G., Campbell R.I., Gibson I., Bernard A., Schulz J., Graf P., Ahuja B. (2016). Design for Additive Manufacturing: Trends, Opportunities, Considerations, and Constraints. CIRP Ann..

[43] Aboulkhair N.T., Simonelli M., Parry L., Ashcroft I., Tuck C., Hague R. (2019). 3D Printing of Aluminium Alloys: Additive Manufacturing of Aluminium Alloys Using Selective Laser Melting. Prog. Mater. Sci..

[44] Girelli L., Tocci M., Gelfi M., Pola A. (2019). Study of Heat Treatment Parameters for Additively Manufactured AlSi10Mg in Comparison with Corresponding Cast Alloy. Mater. Sci. Eng. A.

[45] Spierings A.B., Dawson K., Dumitraschkewitz P., Pogatscher S., Wegener K. (2018). Microstructure Characterization of SLM-Processed Al-Mg-Sc-Zr Alloy in the Heat Treated and HIPed Condition. Addit. Manuf..

[46] Martucci A., Aversa A., Lombardi M. (2023). Ongoing Challenges of Laser-Based Powder Bed Fusion Processing of Al Alloys and Potential Solutions from the Literature—A Review. Materials.

[47] Cabrini M., Lorenzi S., Pastore T., Pellegrini S., Manfredi D., Fino P., Biamino S., Badini C. (2016). Evaluation of Corrosion Resistance of Al–10Si–Mg Alloy Obtained by Means of Direct Metal Laser Sintering. J. Mater. Process. Technol..

[48] Cherry J.A., Davies H.M., Mehmood S., Lavery N.P., Brown S.G.R., Sienz J. (2015). Investigation into the Effect of Process Parameters on Microstructural and Physical Properties of 316L Stainless Steel Parts by Selective Laser Melting. Int. J. Adv. Manuf. Technol..

[49] Saeidi K., Gao X., Zhong Y., Shen Z.J. (2015). Hardened Austenite Steel with Columnar Sub-Grain Structure Formed by Laser Melting. Mater. Sci. Eng. A.

[50] Liverani E., Toschi S., Ceschini L., Fortunato A. (2017). Effect of Selective Laser Melting (SLM) Process Parameters on Microstructure and Mechanical Properties of 316L Austenitic Stainless Steel. J. Mater. Process. Technol..

[51] Röttger A., Geenen K., Windmann M., Binner F., Theisen W. (2016). Comparison of Microstructure and Mechanical Properties of 316L Austenitic Steel Processed by Selective Laser Melting with Hot-Isostatic Pressed and Cast Material. Mater. Sci. Eng. A.

[52] Mertens R., Vrancken B., Holmstock N., Kinds Y., Kruth J.-P., Van Humbeeck J. (2016). Influence of Powder Bed Preheating on Microstructure and Mechanical Properties of H13 Tool Steel SLM Parts. Phys. Procedia.

[53] Krell J., Röttger A., Geenen K., Theisen W. (2018). General Investigations on Processing Tool Steel X40CrMoV5-1 with Selective Laser Melting. J. Mater. Process. Technol..

[54] Vrancken B., Thijs L., Kruth J.-P., Van Humbeeck J. (2012). Heat Treatment of Ti6Al4V Produced by Selective Laser Melting: Microstructure and Mechanical Properties. J. Alloys Compd..

[55] Thijs L., Verhaeghe F., Craeghs T., Humbeeck J.V., Kruth J.-P. (2010). A Study of the Microstructural Evolution during Selective Laser Melting of Ti–6Al–4V. Acta Mater..

[56] Simonelli M., Tse Y.Y., Tuck C. (2014). Effect of the Build Orientation on the Mechanical Properties and Fracture Modes of SLM Ti–6Al–4V. Mater. Sci. Eng. A.

[57] Qiu C., Adkins N.J.E., Attallah M.M. (2013). Microstructure and Tensile Properties of Selectively Laser-Melted and of HIPed Laser-Melted Ti–6Al–4V. Mater. Sci. Eng. A.

[58] Rafi H.K., Karthik N.V., Gong H., Starr T.L., Stucker B.E. (2013). Microstructures and Mechanical Properties of Ti6Al4V Parts Fabricated by Selective Laser Melting and Electron Beam Melting. J. Mater. Eng. Perform..

[59] Brandl E., Palm F., Michailov V., Viehweger B., Leyens C. (2011). Mechanical Properties of Additive Manufactured Titanium (Ti–6Al–4V) Blocks Deposited by a Solid-State Laser and Wire. Mater. Des..

[60] Edwards P., Ramulu M. (2014). Fatigue Performance Evaluation of Selective Laser Melted Ti–6Al–4V. Mater. Sci. Eng. A.

[61] Leuders S., Thöne M., Riemer A., Niendorf T., Tröster T., Richard H.A., Maier H.J. (2013). On the Mechanical Behaviour of Titanium Alloy TiAl6V4 Manufactured by Selective Laser Melting: Fatigue Resistance and Crack Growth Performance. Int. J. Fatigue.

[62] Wycisk E., Siddique S., Herzog D., Walther F., Emmelmann C. (2015). Fatigue Performance of Laser Additive Manufactured Ti–6Al–4V in Very High Cycle Fatigue Regime up to 10^9^ Cycles. Front. Mater..

[63] Cain V., Thijs L., Van Humbeeck J., Van Hooreweder B., Knutsen R. (2015). Crack Propagation and Fracture Toughness of Ti6Al4V Alloy Produced by Selective Laser Melting. Addit. Manuf..

[64] Amato K.N., Gaytan S.M., Murr L.E., Martinez E., Shindo P.W., Hernandez J., Collins S., Medina F. (2012). Microstructures and Mechanical Behavior of Inconel 718 Fabricated by Selective Laser Melting. Acta Mater..

[65] Chlebus E., Gruber K., Kuźnicka B., Kurzac J., Kurzynowski T. (2015). Effect of Heat Treatment on the Microstructure and Mechanical Properties of Inconel 718 Processed by Selective Laser Melting. Mater. Sci. Eng. A.

[66] Deng D., Peng R.L., Brodin H., Moverare J. (2018). Microstructure and Mechanical Properties of Inconel 718 Produced by Selective Laser Melting: Sample Orientation Dependence and Effects of Post Heat Treatments. Mater. Sci. Eng. A.

[67] Zhang D., Niu W., Cao X., Liu Z. (2015). Effect of Standard Heat Treatment on the Microstructure and Mechanical Properties of Selective Laser Melting Manufactured Inconel 718 Superalloy. Mater. Sci. Eng. A.

[68] Li C., White R., Fang X.Y., Weaver M., Guo Y.B. (2017). Microstructure Evolution Characteristics of Inconel 625 Alloy from Selective Laser Melting to Heat Treatment. Mater. Sci. Eng. A.

[69] Silbernagel C., Gargalis L., Ashcroft I., Hague R., Galea M., Dickens P. (2019). Electrical Resistivity of Pure Copper Processed by Medium-Powered Laser Powder Bed Fusion Additive Manufacturing for Use in Electromagnetic Applications. Addit. Manuf..

[70] Lodes M.A., Guschlbauer R., Körner C. (2015). Process Development for the Manufacturing of 99.94% Pure Copper via Selective Electron Beam Melting. Mater. Lett..

[71] Jadhav S.D., Dadbakhsh S., Goossens L., Kruth J.-P., Van Humbeeck J., Vanmeensel K. (2019). Influence of Selective Laser Melting Process Parameters on Texture Evolution in Pure Copper. J. Mater. Process. Technol..

[72] DebRoy T., Mukherjee T., Milewski J.O., Elmer J.W., Ribic B., Blecher J.J., Zhang W. (2019). Scientific, Technological and Economic Issues in Metal Printing and Their Solutions. Nat. Mater..

[73] Olakanmi E.O., Cochrane R.F., Dalgarno K.W. (2015). A Review on Selective Laser Sintering/Melting (SLS/SLM) of Aluminium Alloy Powders: Processing, Microstructure, and Properties. Prog. Mater. Sci..

[74] Read N., Wang W., Essa K., Attallah M.M. (2015). Selective Laser Melting of AlSi10Mg Alloy: Process Optimisation and Mechanical Properties Development. Mater. Des. (1980–2015).

[75] Kempen K., Thijs L., Van Humbeeck J., Kruth J.-P. (2015). Processing AlSi10Mg by Selective Laser Melting: Parameter Optimisation and Material Characterisation. Mater. Sci. Technol..

[76] Sames W.J., List F.A., Pannala S., Dehoff R.R., Babu S.S. (2016). The Metallurgy and Processing Science of Metal Additive Manufacturing. Int. Mater. Rev..

[77] Kok Y., Tan X.P., Wang P., Nai M.L.S., Loh N.H., Liu E., Tor S.B. (2018). Anisotropy and Heterogeneity of Microstructure and Mechanical Properties in Metal Additive Manufacturing: A Critical Review. Mater. Des..

[78] Gibson I., Rosen D., Stucker B., Khorasani M. (2021). Additive Manufacturing Technologies.

[79] Ngo T.D., Kashani A., Imbalzano G., Nguyen K.T.Q., Hui D. (2018). Additive Manufacturing (3D Printing): A Review of Materials, Methods, Applications and Challenges. Compos. Part B Eng..

[80] Bikas H., Stavropoulos P., Chryssolouris G. (2016). Additive Manufacturing Methods and Modelling Approaches: A Critical Review. Int. J. Adv. Manuf. Technol..

[81] Singh S., Ramakrishna S., Singh R. (2017). Material Issues in Additive Manufacturing: A Review. J. Manuf. Process..

[82] Tradowsky U., White J., Ward R.M., Read N., Reimers W., Attallah M.M. (2016). Selective Laser Melting of AlSi10Mg: Influence of Post-Processing on the Microstructural and Tensile Properties Development. Mater. Des..

[83] Li W., Li S., Liu J., Zhang A., Zhou Y., Wei Q., Yan C., Shi Y. (2016). Effect of Heat Treatment on AlSi10Mg Alloy Fabricated by Selective Laser Melting: Microstructure Evolution, Mechanical Properties and Fracture Mechanism. Mater. Sci. Eng. A.

[84] Brandl E., Heckenberger U., Holzinger V., Buchbinder D. (2012). Additive Manufactured AlSi10Mg Samples Using Selective Laser Melting (SLM): Microstructure, High Cycle Fatigue, and Fracture Behavior. Mater. Des..

[85] Martin J.H., Yahata B.D., Hundley J.M., Mayer J.A., Schaedler T.A., Pollock T.M. (2017). 3D Printing of High-Strength Aluminium Alloys. Nature.

[86] Rodrigues T.A., Duarte V., Miranda R.M., Santos T.G., Oliveira J.P. (2019). Current Status and Perspectives on Wire and Arc Additive Manufacturing (WAAM). Materials.

[87] Wu B., Pan Z., Ding D., Cuiuri D., Li H., Xu J., Norrish J. (2018). A Review of the Wire Arc Additive Manufacturing of Metals: Properties, Defects and Quality Improvement. J. Manuf. Process..

[88] Maskery I., Aremu A.O., Simonelli M., Tuck C., Wildman R.D., Ashcroft I.A., Hague R.J.M. (2015). Mechanical Properties of Ti-6Al-4V Selectively Laser Melted Parts with Body-Centred-Cubic Lattices of Varying Cell Size. Exp. Mech..

[89] Murr L.E., Gaytan S.M., Medina F., Martinez E., Martinez J.L., Hernandez D.H., Machado B.I., Ramirez D.A., Wicker R.B. (2010). Characterization of Ti–6Al–4V Open Cellular Foams Fabricated by Additive Manufacturing Using Electron Beam Melting. Mater. Sci. Eng. A.

[90] Gradl P.R., Greene S.E., Protz C., Bullard B., Buzzell J., Garcia C., Wood J., Osborne R., Hulka J., Cooper K.G. (2018). Additive Manufacturing of Liquid Rocket Engine Combustion Devices: A Summary of Process Developments and Hot-Fire Testing Results. Proceedings of the 2018 Joint Propulsion Conference.

[91] Lewandowski J.J., Seifi M. (2016). Metal Additive Manufacturing: A Review of Mechanical Properties. Annu. Rev. Mater. Res..

[92] Mower T.M., Long M.J. (2016). Mechanical Behavior of Additive Manufactured, Powder-Bed Laser-Fused Materials. Mater. Sci. Eng. A.

[93] Uzan N.E., Ramati S., Shneck R., Frage N., Yeheskel O. (2018). On the Effect of Shot-Peening on Fatigue Resistance of AlSi10Mg Specimens Fabricated by Additive Manufacturing Using Selective Laser Melting (AM-SLM). Addit. Manuf..

[94] Afroz L., Qian M., Forsmark J., Li Y., Easton M., Das R. (2025). Fatigue Life of Laser Powder Bed Fusion (L-PBF) AlSi10Mg Alloy: Effects of Surface Roughness and Porosity. Prog. Addit. Manuf..

[95] Baig S., Jam A., Beretta S., Shao S., Shamsaei N. (2025). Fatigue-Defect Criticality in Laser Powder Bed Fused Aluminum Alloys. Theor. Appl. Fract. Mech..

[96] Romano S., Brandão A., Gumpinger J., Gschweitl M., Beretta S. (2017). Qualification of AM Parts: Extreme Value Statistics Applied to Tomographic Measurements. Mater. Des..

[97] Sanaei N., Fatemi A. (2021). Defects in Additive Manufactured Metals and Their Effect on Fatigue Performance: A State-of-the-Art Review. Prog. Mater. Sci..

[98] Beretta S., Romano S. (2017). A Comparison of Fatigue Strength Sensitivity to Defects for Materials Manufactured by AM or Traditional Processes. Int. J. Fatigue.

[99] du Plessis A., Yadroitsava I., Yadroitsev I. (2020). Effects of Defects on Mechanical Properties in Metal Additive Manufacturing: A Review Focusing on X-Ray Tomography Insights. Mater. Des..

[100] Masuo H., Tanaka Y., Morokoshi S., Yagura H., Uchida T., Yamamoto Y., Murakami Y. (2018). Influence of Defects, Surface Roughness and HIP on the Fatigue Strength of Ti-6Al-4V Manufactured by Additive Manufacturing. Int. J. Fatigue.

[101] Pegues J., Roach M., Scott Williamson R., Shamsaei N. (2018). Surface Roughness Effects on the Fatigue Strength of Additively Manufactured Ti-6Al-4V. Int. J. Fatigue.

[102] Greitemeier D., Palm F., Syassen F., Melz T. (2017). Fatigue Performance of Additive Manufactured TiAl6V4 Using Electron and Laser Beam Melting. Int. J. Fatigue.

[103] Solberg K., Berto F. (2019). What Is Going on with Fatigue of Additively Manufactured Metals?. Mater. Des. Process. Commun..

[104] Kahlin M., Ansell H., Basu D., Kerwin A., Newton L., Smith B., Moverare J.J. (2020). Improved Fatigue Strength of Additively Manufactured Ti6Al4V by Surface Post Processing. Int. J. Fatigue.

[105] Frazier W.E. (2014). Metal Additive Manufacturing: A Review. J. Mater. Eng. Perform..

[106] Oliveira J.P., Santos T.G., Miranda R.M. (2020). Revisiting Fundamental Welding Concepts to Improve Additive Manufacturing: From Theory to Practice. Prog. Mater. Sci..

[107] Grasso M., Colosimo B.M. (2017). Process Defects and in Situ Monitoring Methods in Metal Powder Bed Fusion: A Review. Meas. Sci. Technol..

[108] Everton S.K., Hirsch M., Stravroulakis P., Leach R.K., Clare A.T. (2016). Review of In-Situ Process Monitoring and In-Situ Metrology for Metal Additive Manufacturing. Mater. Des..

[109] Tapia G., Elwany A. (2014). A Review on Process Monitoring and Control in Metal-Based Additive Manufacturing. J. Manuf. Sci. Eng..

[110] Yang C., Zhu K., Liu Y., Cai Y., Liu W., Zhang K., Huang J. (2021). A Comparative Study of Fatigue Energy Dissipation of Additive Manufactured and Cast AlSi10Mg Alloy. Metals.

[111] BMW Group (2018). BMW Group Media Information. https://www.press.bmwgroup.com/united-kingdom/article/attachment/T0280549EN/407091.

[112] Kerns J. Metal AM in Automotive: How the Czinger 21C Is Redefining Next-Generation Car Manufacturing. https://www.metal-am.com/articles/metal-3d-printing-in-automotive-how-the-czinger-21c-is-redefining-next-generation-car-manufacturing/.

[113] EOS EOS and Audi Expand Range of Applications for Metal 3D Printing. https://www.eos.info/press-media/press-center/press-releases/2021/cooperation-audi-tooling-2021.

[114] EOS Accelerate Production and Reduce Maintenance: Czech Tool Manufacturer Relies on Additive Manufacturing for Complex Customer Projects. https://www.eos.info/var/assets/01_parts-and-applications/case_studies_applications_parts/_case_studies_pdf/en_cases/cs_m_tooling_innomia_en.pdf.

[115] Pressemitteilung—Fraunhofer IAPT. https://www.iapt.fraunhofer.de/de/presse-medien/presseinformationen/Pressemitteilung_Fiat_Chrysler.html.

[116] Blume M. File:Professional MotorSport World Expo 2024, Cologne (P1031266).jpg. https://commons.wikimedia.org/wiki/File:Professional_MotorSport_World_Expo_2024,_Cologne_(P1031266).jpg.

[117] FraunhoferIAPT File:Bremssattel Bugatti_.jpg. https://commons.wikimedia.org/wiki/File:Bremssattel_Bugatti_.jpg.

[118] BMW Group: Additive Manufacturing Moves Ever Closer to Series Production. https://www.bmwgroup.com/en/news/allgemein/2026/additive-manufacturing.html.

[119] Prova M.O. File:Czinger 21C Chassis Front at Quail 2023.jpg. https://commons.wikimedia.org/wiki/File:Czinger_21C_chassis_front_at_Quail_2023.jpg.

[120] Prova M.O. File:Czinger 21C Chassis Rear at Quail 2023.jpg. https://commons.wikimedia.org/wiki/File:Czinger_21C_chassis_rear_at_Quail_2023.jpg.

[121] Zhao H., Wang A., Li G., Hu Q., Ye C., Shen M., Xiao Y., Liu S., Ji D. (2022). Improving the properties of binder jetted ceramics via nanoparticle dispersion infiltration. Ceram. Int..

[122] (2020). Lightweighting, Materials, Energy Use: How Sustainable Is AM?. Met. Addit. Manuf..

[123] Popiolek A.M., Lebel L.L., Ahrens C.H., de Oliveira Barra G.M., de Souza A.F. (2026). Mechanical Properties of Plastic Parts Produced by Molds Manufactured by Additive Manufacturing Technology (LPBF) with Conformal Cooling Channels. Int. J. Adv. Manuf. Technol..

[124] Xiong J., Zhang G., Hu J., Wu L. (2014). Bead Geometry Prediction for Robotic GMAW-Based Rapid Manufacturing through a Neural Network and a Second-Order Regression Analysis. J. Intell. Manuf..

[125] Ding J., Colegrove P., Mehnen J., Ganguly S., Sequeira Almeida P.M., Wang F., Williams S. (2011). Thermo-Mechanical Analysis of Wire and Arc Additive Layer Manufacturing Process on Large Multi-Layer Parts. Comput. Mater. Sci..

[126] Ding D., Pan Z., Cuiuri D., Li H., Larkin N., van Duin S. (2016). Automatic Multi-Direction Slicing Algorithms for Wire Based Additive Manufacturing. Robot. Comput.-Integr. Manuf..

[127] Aldalur E., Veiga F., Suárez A., Bilbao J., Lamikiz A. (2020). High Deposition Wire Arc Additive Manufacturing of Mild Steel: Strategies and Heat Input Effect on Microstructure and Mechanical Properties. J. Manuf. Process..

[128] Colegrove P.A., Coules H.E., Fairman J., Martina F., Kashoob T., Mamash H., Cozzolino L.D. (2013). Microstructure and Residual Stress Improvement in Wire and Arc Additively Manufactured Parts through High-Pressure Rolling. J. Mater. Process. Technol..

[129] Donoghue J., Antonysamy A.A., Martina F., Colegrove P.A., Williams S.W., Prangnell P.B. (2016). The Effectiveness of Combining Rolling Deformation with Wire–Arc Additive Manufacture on β-Grain Refinement and Texture Modification in Ti–6Al–4V. Mater. Charact..

[130] Guo C., Lin Q., Hu R., Wu S. (2025). Research Progress and Application Scenarios of Wire + Arc Additive Manufacturing: From Process Control to Performance Evaluation. Micromachines.

[131] Jadhav S., Kusekar S., Belure A., Digole S., Mali A., Cheepu M., Mugale M., Alkunte S., Kim D. (2025). Recent Progress and Scientific Challenges in Wire-Arc Additive Manufacturing of Metallic Multi-Material Structures. J. Manuf. Mater. Process..

[132] Dekis M., Tawfik M., Egiza M., Dewidar M. (2025). Challenges and Developments in Wire Arc Additive Manufacturing of Steel: A Review. Results Eng..

[133] Gain S., Veeman D. (2025). A Review on Advances and Challenges in Wire Arc Additive Manufacturing: Process Parameters, Microstructural Evolution and Material Performance across Alloys. J. Alloys Compd..

[134] Sæterbø M., Solvang W.D. (2023). Evaluating the Cost Competitiveness of Metal Additive Manufacturing—A Case Study with Metal Material Extrusion. CIRP J. Manuf. Sci. Technol..

[135] Ferreira B., Brandão F., Borille A., Gonçalves A., Leite M., Ribeiro I. (2025). Assessment of the Technical, Environmental and Economic Trade-Offs in the Early Stage of Metal Additive Manufacturing Adoption. Prog. Addit. Manuf..

[136] Kokare S., Shen J., Fonseca P.P., Lopes J.G., Machado C.M., Santos T.G., Oliveira J.P., Godina R. (2024). Wire Arc Additive Manufacturing of a High-Strength Low-Alloy Steel Part: Environmental Impacts, Costs, and Mechanical Properties. Int. J. Adv. Manuf. Technol..

[137] Laverne F., Segonds F., Anwer N., Le Coq M. (2015). Assembly Based Methods to Support Product Innovation in Design for Additive Manufacturing: An Exploratory Case Study. J. Mech. Des..

[138] Ponche R., Kerbrat O., Mognol P., Hascoet J.-Y. (2014). A Novel Methodology of Design for Additive Manufacturing Applied to Additive Laser Manufacturing Process. Robot. Comput.-Integr. Manuf..

[139] (2023). Additive Manufacturing—Qualification Principles—Requirements for Industrial Additive Manufacturing Processes and Production Sites.

[140] Spears T.G., Gold S.A. (2016). In-Process Sensing in Selective Laser Melting (SLM) Additive Manufacturing. Integr. Mater. Manuf. Innov..

[141] Tang Y., Mak K., Zhao Y.F. (2016). A Framework to Reduce Product Environmental Impact through Design Optimization for Additive Manufacturing. J. Clean. Prod..

[142] Ford S., Despeisse M. (2016). Additive Manufacturing and Sustainability: An Exploratory Study of the Advantages and Challenges. J. Clean. Prod..

[143] Kellens K., Baumers M., Gutowski T.G., Flanagan W., Lifset R., Duflou J.R. (2017). Environmental Dimensions of Additive Manufacturing: Mapping Application Domains and Their Environmental Implications. J. Ind. Ecol..

[144] Faludi J., Bayley C., Bhogal S., Iribarne M. (2015). Comparing Environmental Impacts of Additive Manufacturing vs Traditional Machining via Life-Cycle Assessment. Rapid Prototyp. J..

[145] Paris H., Mokhtarian H., Coatanéa E., Museau M., Ituarte I.F. (2016). Comparative Environmental Impacts of Additive and Subtractive Manufacturing Technologies. CIRP Ann..

[146] Mani M., Lyons K.W., Gupta S.K. (2014). Sustainability Characterization for Additive Manufacturing. J. Res. Natl. Inst. Stand. Technol..

